# Identities, concentrations, and sources of pesticide exposure in pollen collected by managed bees during blueberry pollination

**DOI:** 10.1038/s41598-021-96249-z

**Published:** 2021-08-19

**Authors:** Kelsey K. Graham, Meghan O. Milbrath, Yajun Zhang, Annuet Soehnlen, Nicolas Baert, Scott McArt, Rufus Isaacs

**Affiliations:** 1 Department of Entomology, Michigan State University, 202 CIPS, 578 Wilson Road, East Lansing, MI 48824 USA; 2 Department of Entomology, Cornell University, 4129 Comstock Hall, Ithaca, NY 14853 USA; 3 Present Address: U.S. Department of Agriculture-Agricultural Research Service, Pollinating Insect-Biology, Management, Systematics Research Unit, 1410 N. 800 E., Logan, UT 84341 USA

**Keywords:** Ecology, Agroecology

## Abstract

Bees are critical for crop pollination, but there is limited information on levels and sources of pesticide exposure in commercial agriculture. We collected pollen from foraging honey bees and bumble bees returning to colonies placed in blooming blueberry fields with different management approaches (conventional, organic, unmanaged) and located across different landscape settings to determine how these factors affect pesticide exposure. We also identified the pollen and analyzed whether pesticide exposure was correlated with corbicular load composition. Across 188 samples collected in 2 years, we detected 80 of the 259 pesticide active ingredients (AIs) screened for using a modified QuEChERS method. Detections included 28 fungicides, 26 insecticides, and 21 herbicides. All samples contained pesticides (mean = 22 AIs per pollen sample), with pollen collected from bees on conventional fields having significantly higher average concentrations (2019 mean = 882.0 ppb) than those on unmanaged fields (2019 mean = 279.6 ppb). Pollen collected by honey bees had more AIs than pollen collected by bumble bees (mean = 35 vs. 19 AIs detected at each farm, respectively), whereas samples from bumble bees had higher average concentrations, likely reflecting differences in foraging behavior. Blueberry pollen was more common in pollen samples collected by bumble bees (25.9% per sample) than honey bees (1.8%), though pesticide concentrations were only correlated with blueberry pollen for honey bees. Pollen collected at farms with more blueberry in the surrounding landscape had higher pesticide concentrations, mostly AIs applied for control of blueberry pathogens and pests during bloom. However, for honey bees, the majority of AIs detected at each farm are not registered for use on blueberry at any time (55.2% of AIs detected), including several highly toxic insecticides. These AIs therefore came from outside the fields and farms they are expected to pollinate. For bumble bees, the majority of AIs detected in their pollen are registered for use on blueberry during bloom (56.9% of AIs detected), though far fewer AIs were sprayed at the focal farm (16.7%). Our results highlight the need for integrated farm and landscape-scale stewardship of pesticides to reduce exposure to pollinators during crop pollination.

## Introduction

Flower-visiting insects are important for the production and profitability of pollination-dependent crops^1^ but they may also be exposed to pesticides used to limit losses to insect pests, pathogens, and weeds^2^. These chemicals pose risks to beneficial insects, including bees^3,4^, and so use of pesticides in modern agricultural systems is based on their efficacy against target pests while also limiting risks to non-target organisms. Most insecticides are restricted for use during bloom to mitigate these risks, but there remains limited information on the exposure levels that bees experience in crop systems. Therefore, it is essential to characterize the identity, level, and frequency of exposure, as well as to determine which pesticide combinations bees experience and the probable sources of pesticide residues. With this information, more informed exposure mitigation strategies can be developed.

Previous studies sampling wax, beebread, and honey from inside honey bee colonies revealed exposure to a wide range of pesticides applied to crops^5–7^. Pettis et al.^8^ surveyed honey bees pollinating seven crops and detected high fungicide residues and some insecticides at lower levels. They also found significant pollen collection from outside the crop from weeds and wildflowers. This route of non-crop exposure was also supported by McArt et al.^9^ who found that pesticide risk of colonies in apple orchards was predicted by non-crop pesticide applications or applications to the crop outside of bloom. Simon-Delso et al.’s^10^ data from apiaries also suggests the majority of colony exposure through pollen was not from applications to bee attractive crops, but from weeds or succeeding crops. Additionally, Favaro et al.^11^ found similar fungicide amounts in pollen collected by honey bees in apple orchards during and after bloom, again indicating that exposure outside the focal crop field is important.

Regulatory frameworks for pesticide risk to bees are primarily based on honey bee laboratory and cage studies. While pesticide risk to managed bees is a combination of toxicity and the exposure experienced in the farm setting, field exposure studies are relatively uncommon. Exposure to single active ingredients are rare in agricultural settings, yet toxicity studies on combinations of active ingredients are lacking or they focus on only a few active ingredients^12,13^. There is increasing recognition that the risks to other pollinators including bumble bees should be considered^14,15^, especially because growers also use commercially managed bumble bees as a managed pollinator^16–18^. In particular, field-realistic measurements are needed to understand the complex exposure profiles experienced by different bee species in agricultural settings. Varying foraging behaviors, flight ranges, and flower handling could all combine to affect how much pesticide is collected and transferred to brood by foraging bees^19^. Without measurements of pesticide exposure under typical field conditions, risk assessments have the potential to be under-protective^14,19^.

Additionally, to determine sources of pesticide exposure for honey bees and bumble bees in commercial agriculture, measurements of residues should be combined with records of pesticide applications on farms. By aligning spray records with residues detected, we can better understand how applications on the farm where bees are located or in other external locations contribute to pesticide exposure. Collections across varying management strategies (conventional management, organic, and unsprayed farms) and landscape contexts (high/low natural areas versus agricultural areas) also provides a broader understanding of what conditions can increase exposure. Due to differences in the types of pesticides used on different farms, we expect that organic and unsprayed farms are safer for pollinators, but this assumption may not hold if managed bees are exposed to pesticides from the surrounding landscape. To explore this, we sampled pollen collected by colonies of the honey bee, *Apis mellifera*, and the common eastern bumble bee, *Bombus impatiens,* in blueberry farms and identified pesticide residues at unsprayed, organic, and conventional farms. We focused on the pollen loads on bees returning to colonies to reflect current pesticide exposure from the environment^20,21^ that is largely independent of previous exposure. Exposure in pollen is additionally important, since pollen is fed to larvae and nurse bees and has the potential to affect larval development and the longer-term health of colonies^22^. We used these data to answer the following questions: (1) what pesticides are managed bees exposed to through pollen in blueberry farms; (2) do honey bees and bumble bees vary in their exposure to pesticides in pollen; and (3) how are landscape, crop density, and management strategy related to exposure to pesticides in pollen?

## Methods

### Pollen collection

In 2018 and 2019, we sampled pollen from honey bee colonies at commercial highbush blueberry farms in southwest Michigan. In 2019, we also sampled pollen from bumble bee colonies at the same farms where honey bees were sampled (Fig. S1). Samples collected in 2018 were from 14 spatially separated fields managed by eight growers. Seven of the fields were managed using conventional pest management (conventional), three used organic pest management (organic), and four had no chemical pest management at any time of the year (unsprayed). The average distance between sampled fields was 21.6 km ± 11.8 SE (min = 1.8 km, Fig. S1). This allowed for spatial independence for the most common foraging distances of honey bees (mean distance traveled for pollen = 1078 m^23^) and bumble bees (estimated to be less than 1000 m for *Bombus* spp.^24,25^. In 2019, we sampled at 15 fields managed by six growers. The average distance between sampled fields in 2019 was 16.7 km ± 9.0 (min = 2.0 km). Four of the conventionally managed and three of the unsprayed fields were the same as those used in 2018 (henceforth called longitudinal fields). The others sampled in 2019 were different from 2018 due to loss of farm access, though they were in the same general production region. The additional eight fields resulted in a total of ten conventional fields, five unsprayed fields and no organic fields in 2019. At one conventional field we were only able to sample pollen collected by bumble bees due to lack of access by the beekeeper.

We sampled pollen from three honey bee colonies at each field (N = 42 hives each year). We haphazardly selected queen-right colonies with large clusters from the commercial honey bee colonies that were already present on farms for pollination contracts. The colonies were placed in a typical field perimeter location at each farm and were on average 14.3 m (± 3.1) from the nearest mature blueberry bushes. A 10-frame superior pollen trap (Mann Lake, Hackensack, MN, USA) was installed immediately before the start of blueberry bloom (week of May 14th, 2018, week of May 6th, 2019) and was continuously engaged throughout bloom. We collected pollen from each hive twice in 2018 and three times in 2019. Visits were opportunistic when pesticide restricted entry intervals had expired, and the weather was suitable for bee foraging. Because of some trap failures (e.g. bees finding ways into the hive that avoided the pollen trap), we collected 1.81 ± 0.09 samples per hive in 2018 and 2.31 ± 0.17 in 2019. Samples were brought to the laboratory in coolers with ice and stored at − 20 °C until processing.

During the same week that the honey bees were brought to the fields in 2019, we placed *B. impatiens* colonies (Koppert Biological Systems, Howell, MI, USA) in the field margin of each of the sites described above, as a single Quad containing four separate colonies (N = 60 colonies across 15 farms). Bumble bees were placed away from the honey bees to avoid colony raiding/robbing, spaced on average 185.8 ± 138.1 m away. Each colony contained an estimated 125 workers, a foundress queen, and less than 30% males. A 0.4 L sugar supplement was included at the base of each colony. We placed the Quads on a wood shipping pallet along a wooded edge of the field margin and they were covered with double reflective insulation roll (Reflectix, Markleville, IN, USA) to avoid overheating.

We hand-collected pollen from returning bumble bee foragers four times at each colony during blueberry bloom (May 14–June 6, 2019). Each sampling visit lasted 1.5 h when the bumble bees were actively foraging (08:00–16:00 h, with no precipitation and the temperature above 15 °C). Visits were not always on the same days as visits to honey bee hives. We began each visit by closing the colony doors and trapping returning foragers with pollen on their corbiculae in a queen marking tube (Dadant, Albion, MI, USA). We removed pollen from each corbicula placing one load into a pollen tube for identification and the other from the same bee in a separate tube for pesticide analysis. Collected pollen was stored at − 20 °C until processing.

### Quantification of pesticide residues in pollen

We collected 173 pollen samples from honey bee colonies (76 in 2018 and 97 in 2019). For pesticide residue analysis, we subsampled 5 g from each sample (unique hive and unique day), except for three samples which had less than 5 g of pollen available, in which case smaller amounts were used (small sample weight range = 1.46–3.52 g). The average weight of honey bee pollen samples was 5.02 g ± 0.004 in 2018 and 4.92 g ± 0.058 in 2019. For bumble bee-collected pollen, due to relatively low collection volumes, all pollen for residue analysis collected from the four colonies at each farm (across all visits) was combined into a single sample, for a total of 15 samples (range 2.02–4.95 g, average 3.29 g ± 0.21).

All pollen samples were shipped overnight on dry ice to Cornell University (Ithaca, NY, USA). Frozen pollen samples were extracted by a modified version of the EN 15662 QuEChERS procedure^26^ and screened for 261 pesticides (including some metabolites and breakdown products) by liquid chromatography mass spectrometry (LC–MS/MS) (Table S2). An internal standard solution (d_4_-imidacloprid 0.07 ng/µL; d_10_-chlorpyrifos 0.2 ng/µL: d_7_-bentazon 0.1 ng/µL; d_5_-atrazine 0.02 ng/µL; d_7_-propamocarb 0.1 ng/µL) was used. Detailed methods can be found in the supplemental text. In hive miticide treatments applied by beekeepers (coumaphos and amitraz) were detected but excluded from analyses due to the focus on grower-applied pesticides. For comparisons between pesticide types, only fungicides, insecticides, and herbicides were included. For comparisons between bee species, only the 2019 data were used.

### Identification of pollen

A 10 g (± 0.1 g) subsample of pollen was removed from each honey bee pollen trap collection from 2019 (individual hive on a unique date) and placed in a 50 mL conical tube. Water was then added to the 50 mL mark and the tube was vortexed until the pollen was homogenized (2–3 min). Twenty microliters of the freshly vortexed pollen solution was then added to a slide and allowed to evaporate on a 100 °C hot plate (30–60 s). Fushcin gel was added to the middle of the evaporated sample (~ 3 mm cube) while on the hot plate and allowed to melt prior to the addition of a cover slip and removal from heat.

We analyzed up to ten pollen loads from each bumble bee colony on each sampling date. When fewer than ten pollen loads were collected during that sampling event, we identified as many as were collected (total of 240 pollen loads analyzed, 7.53 ± 0.20 per colony per sampling event). Pollen loads were dehydrated before processing by opening the tubes and placing them in a covered glass container containing desiccant (W. A. Hammond DRIERITE Co. LTD, Xenia, OH) for at least 24 h. After dehydration, we lightly broke up the pollen pellet using the end of a plastic micro-scoop (Disposable Antistatic Polypropylene Sample Transfer Scoops, 2–7 mg Capacity, Lifemode, Synaptenet LLC, Chicago, IL, USA). Approximately 3 mg of pollen was then added to a separate tube, along with 50 μL of distilled water. That tube was vortexed for 15 s or until the pollen was evenly mixed with the water. Twenty-five microliters of the freshly vortexed pollen solution was then added to a slide as described above.

For honey bee pollen, we estimated pollen composition in each sample by counting pollen grains at three haphazardly selected areas on the slide at a magnification of 400×. All pollen grains were identified and counted within the field of vision and a percentage of each pollen type in the sample was then estimated using the pollen counts from the three areas. Unique pollen grains were identified using published guides^27,28^, and cross referencing with Paldat.org and the Isaacs Lab pollen image collection (http://bit.ly/MSUpollen). For bumble bee pollen, each slide was scanned systematically to cover the entire sample, and the percent makeup of each pollen type on the slide was recorded. Here, we only report the percent blueberry pollen, and a detailed analysis of the full pollen collection will be reported separately.

### Landscape classification

We used ArcGIS v10.2.2 (Environmental Systems Research Institute, Redlands, CA, USA) to quantify the proportion of the surrounding landscape in different land uses at multiple spatial scales (500, 1000, and 2000 m), based on the Cropland Data Layer^29^ (CDL; 30 m spatial resolution) provided by the USDA NASS. The maximum scale was selected based on typical foraging distances^23–25^. Since the CDL provides low accuracy in detecting blueberry fields from other fruit crops we merged the CDL with a layer of blueberry fields that was hand digitized and ground-truthed based on National Agriculture Imagery Program aerial images (NAIP: 1 m resolution). The detailed land cover categories (n = 52) were then reclassified into three broad categories of blueberry, other agriculture, and other (all other land cover classifications not included in blueberry or other agriculture). We then determined the percent of blueberry and other agricultural lands across the three scales surrounding the focal fields.

### Data analyses

Analyses and data visualization were performed using R version 3.6.2^30^ and GraphPad Prism 7^31^. All models were checked for overdispersion and zero inflation prior to model selection (function: simulateResiduals, package: DHARMa^32^; simulations = 1000), and model selection was performed by comparison of AICc (function: dredge, package: MuMIn^33^). p values were obtained using the function Anova (package: car^34^) and pseudo-R^2^ values were obtained using the rsquared function (package: piecewiseSEM^35^). Means comparisons were performed using Tukey’s honest significance tests (function: glht, package: multcomp^36^).

#### Active ingredients detected in bee collected pollen

We used a generalized linear mixed effects model (GLMM; function: glmer, package: lme4^37^) with a Poisson distribution to compare the average number of active ingredients (AIs) detected per site between bee species in 2019. Site was included in the model as a random effect. The one site that only had bumble bees was removed for this analysis. To compare the average number of AIs between farm management types, data were separated out by bee species and year, and data were kept at the sample level. We then used GLMMs with site as a random effect to compare between samples from conventional farms, organic farms (2018 only), and unsprayed farms.

#### Pesticide concentrations detected in bee-collected pollen

Pesticide residue amounts were quantified in parts per billion. For detections that were below the limit of quantification but above the limit of detection, the limit of detection was used in data analyses and summaries. Individual AI concentrations were summed by sample to get a total sample pesticide concentration, and then these data were log-transformed to normalize the distribution prior to analyses. We used a linear mixed effects model (LMM) with site included as a random effect to compare the total (log) concentration of pesticides per sample between bee species in 2019. We then separated the data by bee species and year and used the same LMM structure to build separate models to compare differences in concentration per sample between farm management types on the focal farm, and between the three pesticide types (fungicides, insecticides, and herbicides).

#### Comparison of pesticides in pollen between years

We compared sample pesticide concentrations in honey bee collected pollen between years using data from the seven longitudinal fields (N = 38 samples in 2018 and 58 samples in 2019). We first tested for a correlation in average sample pesticide concentration between 2018 and 2019 at the same fields using Pearson’s correlation coefficient (function: cor.test). We then used a linear model with year and farm as interacting factors to compare average sample pesticide concentrations between years.

#### Exposure from the focal field

We collected spray records from collaborating growers, listing the pesticides applied to the focal field. We considered the focal field to be the contiguous blueberry cropping area designated by the collaborating grower where the managed colonies were located, since this area received the same pesticide applications. We then aligned the pesticides detected in the collected pollen with the record of pesticides sprayed on that farm. Only AIs sprayed during bloom before or on the day of detection in each pollen sample were designated as coming from bloom-time pest management. Since bumble bee collected pollen was pooled over time any active ingredients that were detected in the pollen and sprayed during bloom and before sampling of pollen ended were designated as coming from bloom-time focal farm management. We also used spray records from other farms in the area as well as the 2020 Michigan Fruit Management Guide^38^ to determine other active ingredients that could be used on neighboring blueberry farms during bloom. These sources were also used to determine which AIs may have come from farm management outside of bloom (commonly applied pesticides on blueberry farms used at other times of the season), as pesticides can persist on farms well after they have been applied^39–42^. The remaining pesticides detected were not registered for use on blueberry and were therefore assumed to have come from outside blueberry fields. This allowed us to categorize all pesticide detections as either (1) from the bloom-time focal farm management, (2) from neighboring blueberry farm management during bloom, or (3) not from bloom-time blueberry management (either from applications outside of bloom or not from blueberry management).

#### Pollen foraging

We hypothesized that collection of blueberry pollen would increase pesticide exposure in pollen due to pesticide applications during bloom. To test this, we analyzed whether the total concentration of pesticides labeled for use during blueberry bloom (azoxystrobin, boscalid, diuron, fenbuconazole, fluopyram, metconazole, methoxyfenozide, metolachlor, primethanil, propiconazole, and pyraclostrobin) was correlated with percent blueberry pollen collected by bees. We used separate linear models for each bee species (function lm, package: lmer4) with percent blueberry pollen as the factor.

#### Effects of landscape composition on pollen collection and pesticide exposure

We used separate univariate linear models to analyze the correlations of landscape composition (percent blueberry, percent other agricultural lands) at three scales (500, 1000, and 2000 m) with the percent blueberry pollen collected by honey bees and bumble bees in 2019 (function lm, package: lmer4). We also used separate univariate models to analyze the correlations between landscape composition (as above) and pesticide concentrations detected in pollen. We then plotted the relationships of pesticide concentration and percent blueberry in the landscape with 95% confidence intervals (function: geom_smooth(method = lm), package: ggplot2^43^).

Additionally, we analyzed if there was a difference in landscape composition (percent of the landscape in blueberry production or other agricultural lands) across three scales surrounding conventional farms versus unsprayed farms in 2019. This was used to determine whether landscape composition differed between the two management types, because this may confound analysis of how management type affects pesticide exposure from pollen.

## Results

### Active ingredients detected in bee collected pollen

All 188 pollen samples had at least 12 active ingredients detected in each sample, with a maximum of 31 AIs and an average of 22.0 ± 0.3 per sample. Over both years, 80 of the 259 screened pesticide active ingredients were detected in the pollen. These included 28 fungicides, 26 insecticides, 21 herbicides, two miticides, and one rodenticide. We also detected one synthetic antioxidant and one pesticide synergist (Table S1). We detected approximately twice as many AIs in pollen collected by honey bees (68 AIs) in 2019 than in pollen collected by bumble bees (32). All AIs detected in pollen from bumble bees were also collected by honey bees in either 2018 or 2019. Honey bee collected pollen also had significantly more AIs on average detected at each site (35.0 ± 0.9 S.E. AIs per site) compared to bumble bees (18.6 ± 0.6) in 2019 (R^2^m = 0.73; *X*^2^ = 68.2, df = 1, p < 0.001).

**Figure 1:**
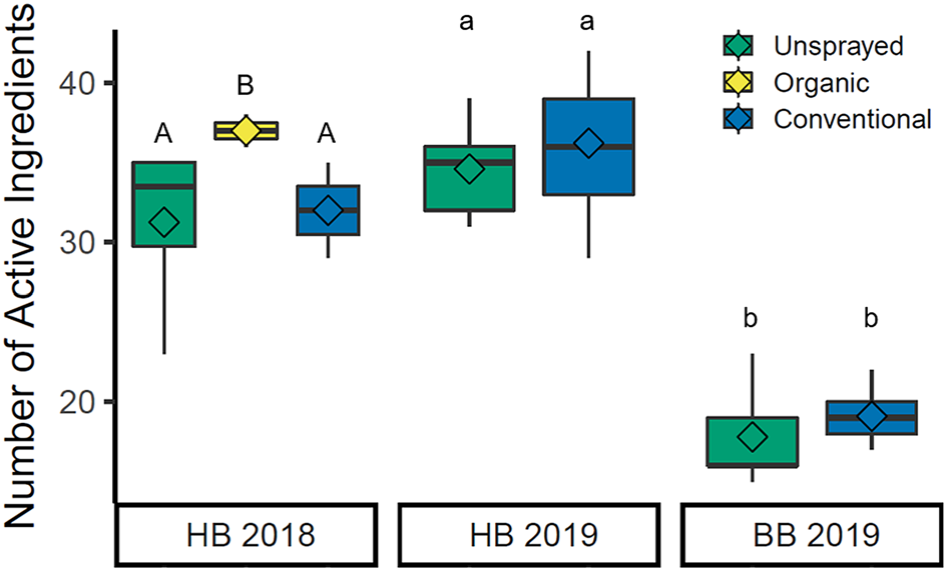
Average number of active ingredients (AIs) detected at each farm. Dark lines indicate the median, diamonds indicate the mean, boxes represent the upper and lower quartile, whiskers indicate the maximum and minimum number of AIs detected. Data are separated by which bee collected the pollen (*HB* honey bee, *BB* bumble bee) and in which year the data were collected. Upper case letters indicate significant differences within the 2018 data, and lower case letters indicate significant differences within the 2019 data. Graph created in R^30^ v3.6.2 with the package ggplot2^43^.

Farm management strategy (conventional, organic, or unsprayed) influenced the average number of pesticides detected in pollen samples collected from honey bees in 2018, but not in 2019 (Table 1, Fig. 1). For honey bee pollen in 2018, samples from organic farms had more individual pesticide AIs detected on average than that from conventional (Tukey’s HSD: p = 0.031) or unsprayed farms (Tukey’s HSD: p = 0.027) (Table 2, Fig. 1). In 2019, there was no significant difference in the average number of AIs found at conventional or unsprayed farms for either honey bees (p = 0.90) or bumble bees (p = 0.58) (Table 2, Fig. 1).

**Table 1:** Summary table of pesticide detections from pollen collected from honey bees (HB) or bumble bees (BB) on blueberry farms in 2018 and 2019.

Bee	Year	Field management	# of AIs	AIs per sample (mean ± S.E.)	Average pesticide conc. (ppb ± S.E.)	Fungicide(s) with highest frequency (AI, % samples)	Max. fungicide (AI, ppb)	Insecticide(s) with highest frequency (AI, % samples)	Max. insecticide (AI, ppb)	Herbicide(s) with the highest frequency (AI, % samples)	Max. herbicides (AI, ppb)
HB	2018	Unsprayed	48	21.6 ± 0.5^B^	85.8 ± 11.8^b^	*Fluopyram*, 100%	Carbendazim, 80.2	Clothianidin, imidacloprid 95.2%	Carbaryl, 49.6	Atrazine, *metolachlor*, 100%	*Metolachlor*, 36.9
		Organic	46	26.0 ± 0.5^A^	180.8 ± 29.6^ab^	*Azoxystrobin, boscalid,* difenoconazole, *fenbuconazole, fluopyram, pyraclostrobin,* 100%	*Metconazole*, 99.3	Carbaryl, imidacloprid *methoxyfenozide,* 100%	*Methoxyfenozide*, 285.4	Atrazine, *metolachlor,* 100%	Dimethenamid, 31.5
		Conventional	54	21.2 ± 1.1^B^	286.2 ± 34.1^a^	*Azoxystrobin, pyraclostrobin* 100%	*Azoxystrobin*, 585.8	Imidacloprid, 100%	*Methoxyfenozide*, 285.9	Atrazine, *metolachlor,* 100%	*Metolachlor*, 54.4
HB	2019	Unsprayed	55	20.5 ± 0.5^A^	287.3 ± 81.7^b^	*Azoxystrobin*, *fluopyram* 100%	Carbendazim, 2333.2	Chlorpyrifos, 97.1%	Carbaryl, 93.2	Atrazine, *metolachlor,* 100%	Atrazine, 320.5
		Conventional	61	20.6 ± 0.7^A^	770.0 ± 139.3^a^	*Azoxystrobin, fluopyram*100%	Carbendazim, 5753.6	Chlorpyrifos, 96.8%	*Methoxyfenozide*, 277.6	Atrazine, *metolachlor*, 100%	*Diuron*, 475.6
BB	2019	Unsprayed	29	17.8 ± 1.5^A^	330.3 ± 116.8^b^	*Azoxystrobin, boscalid,* carbendazim*, fenbuconazole, fluopyram, pyraclostrobin,* thiophanate methyl*,* 100%	*Fenbuconazole*, 554.5	Chlorpyrifos, 100%	Chlorpyrifos, 272.7	Atrazine, *metolachlor,* 100%	*Metolachlor*, 30.3
		Conventional	31	19.1 ± 0.5^A^	1708.2 ± 226.6^a^	*Azoxystrobin, boscalid,* cyprodinil, *fenbuconazole, fluopyram*, thiophanate methyl, 100%	*Boscalid*, 1757.6	Chlorpyrifos, *methoxyfenozide*, imidacloprid 100%	*Methoxyfenozide*, 1406.2	Atrazine, *diuron, metolachlor,* 100%	*Diuron*, 375.2

Number of active ingredients (AIs) detected includes all AIs detected within a farm type (unsprayed, organic, or conventional) in each year. Average AIs and pesticide concentrations were calculated by averaging the totals between individual samples. Maximum detections are the highest detections of AIs within each group of samples. AIs in italics are registered for use in highbush blueberry during bloom. Superscript letters indicate significant differences within a year/bee combination.

**Table 2:** Statistical results from generalized linear mixed effects models (Poisson distribution) used to test the effect of farm management on number of active ingredients (AIs) detected bee collected pollen, and results from linear mixed models used to test the effects of farm management on pesticide concentrations in pollen, as well as differences in pesticide types (fungicides, insecticides, herbicides) found in the pollen.

	Factor	R^2^m	Test statistic	df	p value
**Honey bees 2018**					
Num. of AIs	Farm management	0.125	*X*^2^ = 0.527	2	**0.014**
Avg. concentration	Farm management	0.324	*X*^2^ = 10.017	2	**0.007**
	Pesticide type	0.322	*X*^2^ = 161.99	2	** < 0.001**
**Honey bees 2019**					
Num. of AIs	Farm management	0.000	*X*^2^ = 0.001	1	0.972
Avg. concentration	Farm management	0.167	*X*^2^ = 6.037	1	**0.014**
	Pesticide type	0.489	*X*^2^ = 339.6	2	** < 0.001**
**Bumble bees 2019**					
Num. of AIs	Farm management	0.017	*X*^2^ = 0.250	1	0.617
Avg. concentration	Farm management	0.620	F_1,14_ = 21.173		** < 0.001**
	Pesticide type	0.398	F_2,43_ = 13.886		** < 0.001**

### Pesticide concentrations detected in bee collected pollen

Across both years and bee species, the average concentration of all detected pesticides in individual pollen samples was 477.9 ppb ± 57.1. Contrary to patterns observed in the number of AIs detected, we found significantly higher average concentrations of pesticides in pollen collected from bumble bees (1243.4 ppb ± 231.4) compared to honey bees in 2019 (577.7 ppb ± 95.8) (R^2^m = 0.023; *X*^2^ = 50.94, df = 1, p < 0.001).

Bees on conventionally managed farms had higher concentrations of pesticides in their pollen (Table 1). For honey bee-collected pollen in 2018, conventionally managed farms had significantly higher average concentrations of pesticides compared to unsprayed farms (Tukey’s HSD: p = 0.004), with no significant difference between organic and conventional farms (Tukey’s HSD: p = 0.60) and no significant difference between unsprayed and organic farms (Tukey’s HSD: p = 0.19) (Table 2). For both honey bee and bumble bee collected pollen in 2019, samples from conventional farms had significantly higher average concentrations of pesticides compared to unsprayed farms (Honey bees: p = 0.014. Bumble bees: p < 0.001) (Table 2).

**Figure 2:**
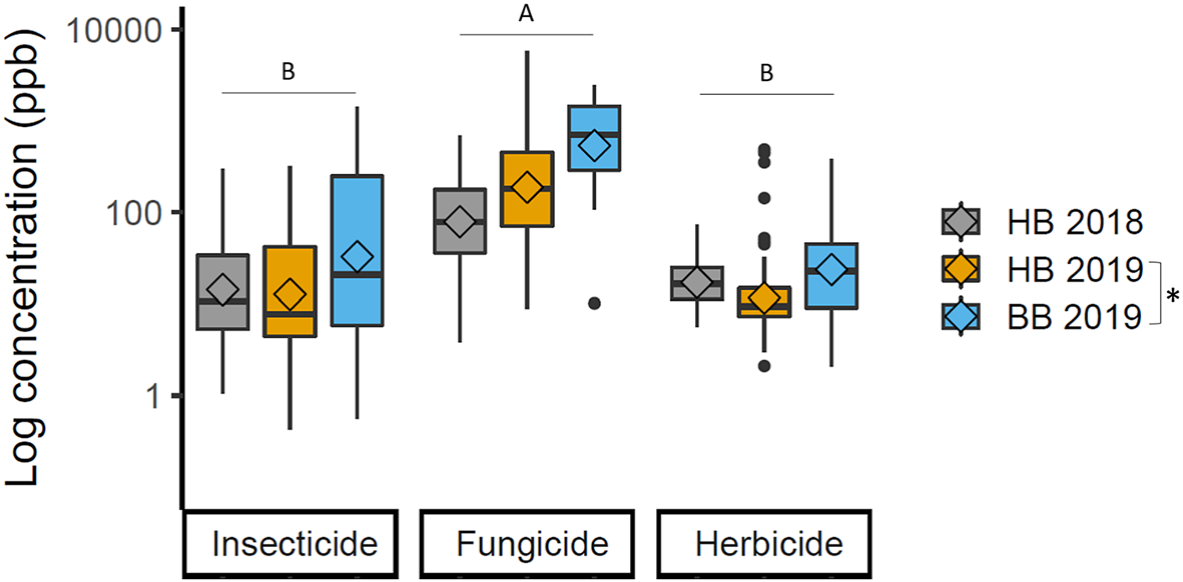
Concentrations of pesticides detected in bee-collected pollen from colonies. Each data point represents the concentration of an active ingredient found in an individual sample. Pesticides were detected in pollen collected from honey bees in 2018 (grey), pollen collected from honey bees in 2019 (yellow), and pollen collected from bumble bees in 2019 (blue). Dark lines indicate the median, diamonds indicate the mean, boxes represent the upper and lower quartile, whiskers indicate the maximum and minimum concentration detected (besides outliers), and the dots represent outliers. Letters indicate significant differences between the pesticide types and this pattern was consistent when all samples were combined for analyses, or when samples were separated out by bee and year for analyses. Graph created in R^30^ v3.6.2 with the package ggplot2^43^.

Across all samples, fungicides were detected at significantly higher average concentrations (393.6 ppb ± 53.6) compared to insecticides (56.3 ppb ± 10.9; Tukey’s: p < 0.001) and herbicides (26.5 ppb ± 4.5; Tukey’s: p < 0.001) (R^2^m = 0.402; *X*^2^ = 456.5, df = 2, p < 0.001) (Fig. 2). There was no significant difference between average concentration of herbicides and insecticides (Tukey’s: p = 1.00) (Fig. 2).

Fungicides also represented the highest individual detections in the pollen samples (Table 1). Of the top ten highest detections across all samples, nine of them were fungicides (range 1365.9–5753.6 ppb). The other was an insecticide (methoxyfenozide, 1406.2 ppb) (Table 1). The highest detection of an herbicide (diuron) was 34th in overall detection ranking at 475.6 ppb (Table 1).

### Comparison of pesticides in pollen between years

We found that patterns of pesticide detection in honey bee-collected pollen were highly variable between farms and across years, with no consistent trends detected at the seven longitudinal farms. Sixty-six total AIs were detected in honey bee collected pollen at the longitudinal farms over the 2 years of sampling. Of those, forty-two active ingredients were detected in both years, while ten AIs were only detected in 2018 and 14 AIs were only detected in 2019. Additionally, the average concentration of pesticide residues detected in honey bee pollen at a given farm was not significantly correlated between years (Pearson’s r = 0.45, t = 1.12, df = 5, p = 0.31), indicating that exposure is not consistent year to year at individual farms. While average pesticide concentrations were higher at the longitudinal farms in 2019 (428.1 ppb ± 91.7) compared to 2018 (203.3 ppb ± 28.9), this was largely driven by one farm (Fig. S2, Farm 4). Farm was significant in the linear model (R^2^ = 0.56; F_7,83_ = 10.39, p < 0.001), while Year was not (F1_,83_ = 3.54, p = 0.06). Though the interaction of Year and Farm was significant in the model (Year*Farm: R^2^ = 0.56; F_5,83_ = 5.31, p < 0.001), making it hard to interpret the role of individual factors.

### Source of pesticides in pollen

**Figure 3:**
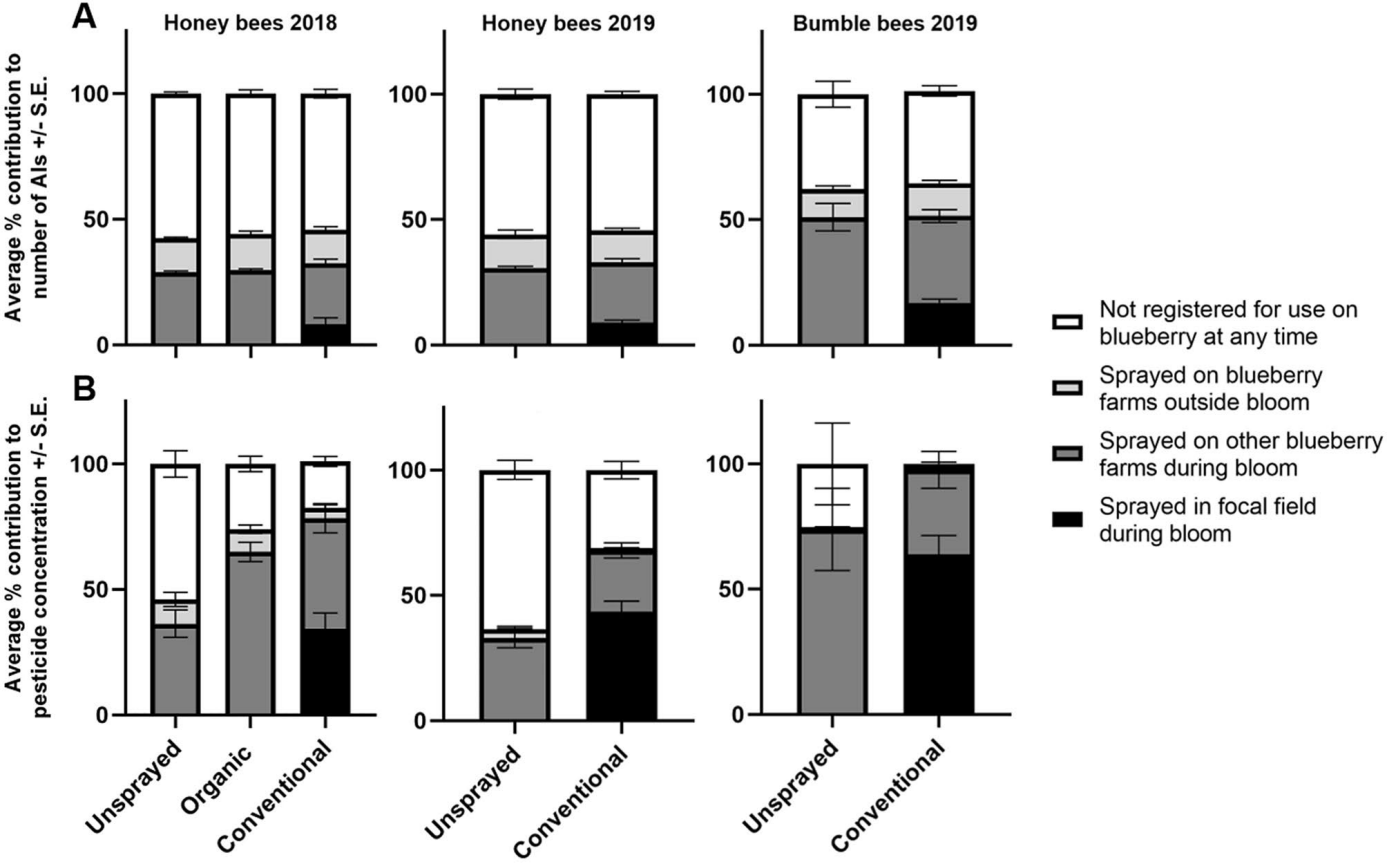
Average percent contribution of pesticides to the (**A**) number of active ingredients (AIs) detected at a site, and (**B**) overall sample concentration. Contributions were determined by spray records and registration status. Active ingredients are separated into those that were either applied on the focal field during bloom (black), registered for use on blueberries during bloom but not sprayed in the focal fields (dark grey), registered for use on blueberries outside bloom (light grey) or not registered for use on blueberries at any time (white). Graph created in GraphPad Prism 9^31^.

Based on spray records and management guides, of the 80 AIs detected in bee-collected pollen, 12 AIs (15% of AIs detected) are commonly applied to blueberry fields during bloom (Table S1), and even fewer were actually sprayed on focal fields; nine AIs were applied to focal fields in 2018 and seven in 2019. For honey bees, the majority of AIs detected are not registered for use on blueberry at any time. This was the case in both 2018 and 2019, and for all field management types, with AIs not registered for use in blueberry averaging between 54.1 and 57.5% of the AIs detected at each farm (Fig. 3A). Far fewer of the AIs were sprayed on the focal farms, 8.4% of AIs detected on conventional farms in 2018 and 8.9% in 2019. For bumble bees, AIs not registered for use on blueberries accounted for less of the AIs detected, averaging 36.8% of the AIs detected at conventional farms and 37.8% at unsprayed farms (Fig. 3A), with the majority of AIs either being sprayed on the focal farm or likely other blueberry farms during bloom (conventional—51.5% of AIs, unsprayed—51.0%). However, only 16.7% of AIs detected in bumble bee pollen collected from colonies on conventional farms were those sprayed at the focal farm.

Eight active ingredients were detected in over 90% of all samples—atrazine, azoxystrobin, boscalid, chlorpyrifos, fluopyram, imidacloprid, metolachlor, and pyraclostrobin (Table S1). Only three of these were applied during bloom on the focal farms in 2018 and 2019: the fungicides azoxystrobin, boscalid and pyraclostrobin. In 2019, fluopyram was also applied. Although not reported in the application history of focal farms, metolachlor is commonly applied in conventionally managed blueberry fields for weed control. Atrazine and chlorpyrifos were not applied at any time in blueberry fields, and imidacloprid was only applied on some non-focal farms after bloom. The frequency of AIs detected in sampled varied somewhat by bee species and focal farm management (Table 1), though fungicides applied for management of blueberry pathogens during bloom were common across all sample types. Conversely, for insecticides, AIs not registered for use on blueberry farms during bloom were more common, including carbaryl, chlorpyrifos, clothianidin, and imidacloprid. Imidacloprid is registered for use on blueberry after bloom, carbaryl is registered for use outside bloom though is rarely used, and the others are not registered for use at any time on blueberry (Table S1). Methoxyfenozide, which is used for blueberry pest control during bloom was also common (Table 1). For herbicides, atrazine and metolachlor were found in every sample (Table 1), and while metolachlor is commonly used on blueberry farms during bloom, atrazine is not registered for use at any time (Table S1).

The overall highest concentrations of pesticide residues were associated with blueberry pest management during bloom. Six pesticides had detections with concentrations that were above the upper limit of linearity: boscalid (10 detections above ULOL), pyraclostrobin (5 detections above ULOL), pyrimethanil (5 detections above ULOL), azoxystrobin (3 detections above ULOL), carbendazim (3 detections above ULOL), and methoxyfenozide (1 detection above ULOL) (see Table S2 for ULOL concentrations). Of these active ingredients, all were applied in conventional blueberry fields used in this study during bloom (though not on all farms), except carbendazim, which is not used in blueberry pest management (Table S1).

The contribution of AIs from blueberry pest management to the overall sample concentration varied based on focal farm management and bee species but followed somewhat similar trends as the contribution to the number of AIs (Fig. 3B). For honey bees at unsprayed fields, the majority of the overall sample pesticide concentration came from AIs not registered for use on blueberry (conventional/organic) at any time of the year, with much less contribution from blueberry AIs applied during bloom or post-bloom on conventional fields (Fig. 3B). In contrast, AIs sprayed on conventional blueberry farms during bloom contributed the most to the pesticide concentrations for bumble bees on unsprayed farms, with exposure likely happening at neighboring conventional blueberry farms. Similar exposure occurred for honey bees at organic farms.

For honey bees at conventional farms, the contribution of AIs to the sample concentration was split between AIs that were sprayed on the focal farm, those that were likely sprayed on neighboring conventional farms, and AIs that are not registered for use on blueberry. Much lower contribution came from AIs used on blueberry farms post-bloom. In contrast, for bumble bees the majority of the pesticide concentration in samples came from AIs sprayed on the focal farm, with the rest primarily being AIs sprayed on neighboring blueberry farms during bloom. Much less contribution came from AIs not registered for use on blueberry or those sprayed on blueberry post-bloom (Fig. 3B).

For pollen collected by honey bees in 2018 and bumble bees in 2019, the majority of high detections came from AIs that were either sprayed on the focal farm during bloom or likely from neighboring blueberry farm management during bloom (Table 1). However, for honey bees in 2019, the highest detections came from an AI not registered for use in blueberry pest management at any time of the year. Carbendazim was the highest detection at both conventional (5753.6 ppb) and unsprayed (2333.2 ppb) farms in 2019 (Table 1). Carbendazim and thiophanate-methyl, of which carbendazim is a metabolite, are AIs of fungicides not registered for use in blueberries so it is assumed these residues came from farms growing other crops. Conversely, for bumble bees at the same farms, the highest detections (top 20 highest detection concentrations for bumble bees) were all AIs of products registered for use in blueberries during bloom, including the fungicide boscalid (highest: 1757.6 ppb) and the insecticide methoxyfenozide (1406.2 ppb) (Table 1).

### Blueberry pollen collection

On average, pollen trapped from honey bee colonies in 2019 had 1.8% ± 3.2 blueberry pollen, while pollen collected from bumble bees had 25.9% ± 3.2 blueberry pollen. Blueberry pollen was collected from honey bee pollen traps at eight out of the 14 farms in 2019. At these eight farms, blueberry pollen made up between 0.04 and 16.7% of the total pollen collected. Pollen collected by bumble bees included blueberry pollen in all 15 farms in 2019, ranging from 3.9% of total pollen gathered to 45.6%.

Although the amount of blueberry pollen explained only 6% of the variation in concentration of pesticides used during blueberry bloom, there was a positive correlation between pesticide concentration and the amount of blueberry pollen collected from honey bee pollen traps (R^2^ = 0.06; F_1,95_ = 5.54, p = 0.02). This relationship was not significant for bumble bees (R^2^ = 0.11; F_1,14_ = 1.53, p = 0.24).

### Effects of landscape composition on pollen collection and pesticide exposure

**Figure 4:**
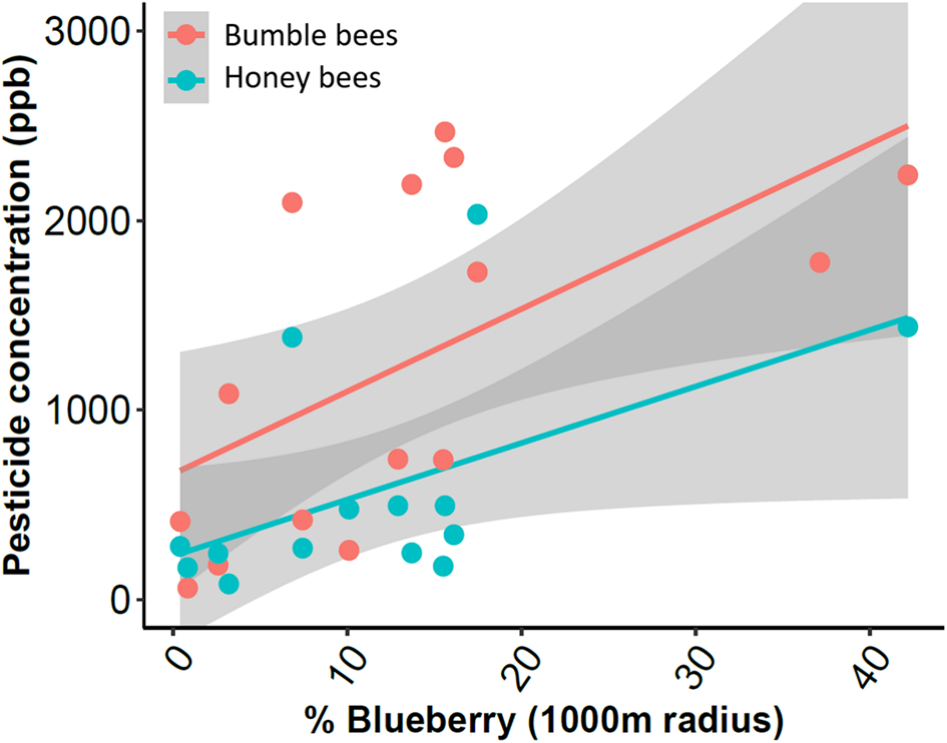
Relationships between percent of the landscape in blueberry production within 1000 m of bee colonies and the concentration of pesticides detected in pollen collected by bumble bees (red) and honey bees (blue). Lines indicate smoothed linear regression lines with 95% confidence intervals. Graph created in R^30^ v3.6.2 with the package ggplot2^43^.

Across all sampled fields in 2019, the average (± SE) percent blueberry fields in the surrounding landscape was 22% ± 5 at the 500 m scale, 12% ± 3 at 1000 m, and 8% ± 2 at 2000 m. The average amount of blueberry pollen collected by honey bees increased with the percent of blueberry in the landscape at 500 m (R^2^ = 0.37, F_1,12_ = 7.18, p = 0.02) and at 1000 m scale (R^2^ = 0.59, F_1,12_ = 16.44, p < 0.01), but not at 2000 m. No significant relationship was found between the amount of blueberry pollen collected by bumble bees and percent blueberry at the three tested spatial scales (p > 0.05) (Fig. S3). There was a significant positive correlation between percent blueberry in the landscape and pesticide concentration in the pollen collected by bumble bees (500 m: R^2^ = 0.49; F_(1,14)_ = 12.70, p = 0.003; 1000 m: R^2^ = 0.34; F_(1,14)_ = 6.79, p = 0.022) (Fig. 4). Pesticide concentration detected in honey bee collected pollen was only significantly correlated with percent blueberry area at the 1000 m scale (R^2^ = 0.34, F_1, 13_ = 6.13, p = 0.029) and not at the 500 m or 2000 m scales (Fig. 4; Fig. S3). There was also no significant relationship between pesticide concentration in pollen collected by both bee species and percent blueberry located at 2000 m scale, as well as percent other agriculture in the landscape across all scales (p > 0.05).

There was a significantly larger average percent of blueberry located within the 500 m scale landscapes surrounding conventional farms (N = 10, 31.4% ± 5.6) compared to unsprayed farms (N = 5, 7.7% ± 3.5) in 2019 (R^2^ = 0.38; F_1,14_ = 7.83, p = 0.015), but no significant difference was found at the larger scales (1000 m and 2000 m; p > 0.05). There was also no significant difference in percent other agricultural lands between the two farm management types at any spatial scale (p > 0.05).

## Discussion

The exposure of managed bees to pesticides via pollen was found to be widespread during blueberry pollination, with a wide diversity of active ingredients, including insecticides, fungicides, and herbicides. Honey bees and bumble bees both collected pesticides targeting different agricultural pests, with AIs detected at the highest frequency being the fungicides azoxystrobin, boscalid, fluopyram, and pyraclostrobin used to prevent fruit rots. Other pesticides were also detected at high frequency, including the herbicide atrazine and the insecticides imidacloprid and chlorpyrifos, which were not applied to the blueberry fields adjacent to the colonies during bloom. Our results demonstrate that pesticides are widespread and persistent in and around this crop system and are collected by bees as they forage across the landscape beyond the farm fields they are placed at for pollination. All samples contained multiple AIs and groups of AIs were detected at very high frequencies, highlighting the importance of understanding the combined effects of field-realistic doses of pesticides on bee health^44,45^.

Pesticide concentrations were higher in pollen collected from colonies in conventional fields and in fields surrounded by a greater proportion of blueberry production. These results align with previous studies that have explored bee exposure in crop systems where pesticides are applied to crops during bloom^8,9,46^. We also found no significant difference in the number of active ingredients between unsprayed and conventional farms, indicating that bees are collecting many of the residues from outside the focal crop fields. The greater number of pesticides detected in honey bee pollen also likely reflects their larger foraging range^19^, which increases their likelihood of contacting novel sources of exposure.

We found much more blueberry pollen in samples from bumble bees compared to honey bees. Honey bees are not well adapted to collecting blueberry pollen due to the poricidal anthers of blueberry flowers, whereas bumble bees use buzz pollination to aid in pollen release^47^. Honey bees also only pack blueberry pollen into small loads on their corbiculae, which may not be dislodged by traps^48^, possibly resulting in an underrepresentation of blueberry pollen in the analyzed samples. However, other research shows low rates of blueberry pollen collection by honey bees even when pollen across the entire bee is considered and pollen is removed from the corbiculae similar to our methods of removing pollen loads from bumble bees^49^, and the large difference in blueberry pollen collection between the species is similar to patterns in other studies^8,47,50^. Although overall collection of blueberry pollen was low for honey bees, the proportion of blueberry pollen collected was positively correlated with the amount of blueberry in the landscape within the 500–1000 m scale, suggesting that honey bees will collect blueberry pollen as abundance increases in the landscape. No such relationship was seen for bumble bees, either because their foraging decisions are more linked to local resource availability, or because they are preferentially collecting blueberry pollen.

The concentration of blueberry pest management-related pesticides in pollen samples was not strongly correlated with proportion of blueberry pollen for either bee species, though it was statistically significant for honey bees. The lack of correlation for bumble bees may indicate that other flowering plants contribute to exposure to bloom-time pest management AIs, perhaps through pesticide drift onto flowering weeds, or that residues from blueberry pollen are highly variable which could obscure our ability to determine the source. Both species interact with blueberry flowers when collecting pollen and/or nectar^47,51^, so some of the residues detected in this study may come from contact with the corolla. Pesticides from flower handling could then be groomed into the pollen loads packed on their corbiculae. Both bee species will visit a variety of pollen resources and mix pollens within a load, though load mixing tends to be less common in honey bees^49,52^. Therefore, pollen mixing may also obscure our ability to determine the source(s) of exposure.

Pesticide exposure was higher in conventional fields than unsprayed fields and it increased with larger blueberry acreage surrounding the colonies. Conventional management and percent blueberry in the landscape, however, were correlated at the 500 m landscape scale, making it difficult to determine the effect of either factor alone at this scale. Although, landscape composition was significantly correlated with exposure for both species at the 1000 m scale, where farm management and landscape composition were not significantly correlated. Therefore, the data suggest that both farm management and landscape context contribute to exposure. But given that collection of blueberry pollen was not highly correlated with pesticide exposure, identification and characterization of other routes of exposure within blueberry fields such as from weeds and surrounding vegetation^53^ will be important for developing strategies to mitigate exposure.

During bloom, protection of blueberries against mummy berry, botrytis, and other diseases is achieved using fungicides that prevent spores from infecting via the stigma^54,55^. It was therefore unsurprising that high concentrations of fungicides used for bloom-time blueberry pest control were found in the pollen of both species of bees. We found higher residue concentrations of azoxystrobin, boscalid, fenbuconazole, metconazole, and pyraclostrobin than other fungicides, reflecting their common use in blueberry management. Indeed, most of the highest detections found in pollen samples were of fungicides used to control blueberry diseases, and all samples except one had at least one detection of these AIs (99% of samples). This included samples from bees adjacent to organic or unsprayed farms where these AIs are not used, indicating that bees are foraging at nearby conventional farms where these products are applied. Carbendazim also had several high detections and was detected in 91% of samples. Thiophanate-methyl, of which carbendazim is a metabolite, is not used in blueberry pest management, but is labeled for use on apples, stone fruits, strawberries, and soybeans, all of which are also grown in this production region. This provides further evidence that foraging of bees outside the focal crop field contributes to their pesticide exposure^10^.

We also found high concentrations of the insect growth regulator methoxyfenozide which is used at some farms during bloom to prevent infestation of berries with cranberry and cherry fruitworms^56^. Methoxyfenozide was detected in 80% of samples and represented the majority of high insecticide detections. This pesticide has previously been found to have sublethal effects in honey bees such as reduced foraging and reduced ability to thermoregulate when added to pollen provisions at concentrations of 100–200 ppb^57^, and we found 17 samples with detections over 100 ppb, including from conventional and organic fields and in both honey bee and bumble bee collected pollen. The average methoxyfenozide sample concentration for honey bees on conventional fields was 41.4 ppb ± 11.2 in 2019, and 339.1 ppb ± 165.0 for bumble bees, likely reflecting the greater fidelity of bumble bees to foraging on blueberry. Carbaryl also represented several high detections and was found in 58% of samples. This insecticide is very rarely applied to blueberry fields in this region^58^ and is not registered for use during bloom. It is applied to tree fruits such as apples, pears, and cherries, where post bloom applications are likely to overlap in timing with blueberry bloom in nearby farms, again providing evidence of exposure from outside the focal crop. The most commonly detected insecticide was chlorpyrifos, found in 89% of pollen samples. This has been frequently detected in honey bee pollen in other regions^5,6,42,59^, and concentrations of chlorpyrifos were sometimes high in our samples. This insecticide is not labeled for use in blueberry at any time and it is restricted on blooming crops due to negative effects on honey bees^60^. The effects on queen production can be worsened when exposure is in combination with the fungicide Pristine (combination of boscalid and pyraclostrobin)^61^, which we also detected at high frequencies in our pollen samples. Chlorpyrifos is labeled for use as a lower trunk spray for apple, cherry, and grape as well as for use in field crops and other less common crops to this production region. Since these applications are not made to bee-attractive flowering crops, our results indicate that residues in pollen are from outside the focal field, from agricultural weeds and other flowering plants.

Comparison of residues detected in honey bees over 2 years at the longitudinal farms shows that pesticide exposure measures for bees within the same farm are highly variable from year to year. This may reflect grower’s adoption of IPM programs that respond to variable pathogen and insect pest risk, resistance management that emphasizes rotating chemical classes, or given the results described above it could also reflect changes in neighbor practices. We were also not able to standardize the timing of sample collection relative to application, and this may also drive some of this variability. Additionally, we did not test for biological pesticides such as *Bacillus thuringiensis* and *Chromobacterium subtsugae* that may be used for insect control and could cause further variability between years and among farms.

All samples contained pesticides not labeled for use on blueberries, indicating that substantial pesticide exposure through pollen is occurring from areas outside the grower’s control. Some of these detections were in colonies placed in unsprayed farms or organic farms where the bees forage far beyond the boundary of land certified for organic production. This further highlights the need for countries and states to develop and implement managed pollinator protection plans to guide widespread reduction of pesticide use in areas beyond the limits of farmland to limit exposure from all sources^62^. A lot of current messaging regarding minimizing pesticide exposure to pollinators is directed to growers who rent or purchase bees for pollination. While this targeted education is important for reducing exposure on the focal farms, our data show that additional extension and education resources are needed for nearby growers and land managers to reduce the large burden of pesticides that are obtained on neighboring properties.

Our data also document differences in exposure between species of bees, even while at the same site However, while toxicity information is largely available for honey bees, it is much more limited for bumble bees, plus the majority of information is on toxicity of direct topical applications to bees rather than exposure through bee bread consumed by larvae^63^. Expanding this knowledge base is a priority for enabling more comprehensive and relevant risk assessments^15^. There is also a need to better understand pesticide exposure to wild bees in pollination-dependent crops. Honey bee colonies are removed by beekeepers after crop bloom to areas with greater flower abundance and diversity and with lower pesticide risk^64^, whereas wild bees remain. To deliver on the promise of integrated pest and pollinator management strategies^65^ balancing pest management and pollinator protection goals, the risk of pesticides to all types of bees must be better understood.

We have documented that managed bee colonies brought to farms during bloom to provide pollination services are exposed to a wide range of pesticides, and that pesticide exposure through pollen is variable between years, landscapes, farming practices, and bee species. In future risk analyses, these data can be used with the relative toxicities of these AIs to bees for calculations of risk^9^. The variability in exposure that we detected under different management and landscape contexts can also be used to better inform risk management recommendations on farm with similar conditions. Additionally, more exposure studies such as this are needed across diverse agricultural landscapes to develop a more complete understanding of conditions that results in substantial pesticide exposure for bees.

## Permissions

This work was conducted on blueberry farms with the permission of cooperating land owners and with commercial honey bee colonies with permission from cooperating beekeepers. We would like to thank all cooperators for their ongoing support of research initiatives.

Bees collected pollen naturally from the environment and collection of pollen from bees was done non-destructively without any harm to the bees, and so there was no institutional or governmental restrictions on these collections.

## Supplementary Information

Supplementary Information 1.
