# Supplementary material for: Identities, concentrations, and sources of pesticide exposure in pollen collected by managed bees during blueberry pollination
Source: Sci Rep. 2021 Aug 19;11:16857. doi: 10.1038/s41598-021-96249-z (PMC8377133; doi:10.1038/s41598-021-96249-z)
Supplement: Supplementary file 1 — Supplementary Information 1. [file 41598_2021_96249_MOESM1_ESM.pdf]

SUPPLEMENTAL: Identities, concentrations, and sources of pesticide exposure in pollen collected by managed bees during blueberry pollination

Kelsey K. Graham[1,3*], Meghan O. Milbrath[1], Yajun Zhang[1], Annuet Soehnlen[1], Nicolas Baert[2], Scott McArt[2], and Rufus Isaacs[1]

1. Department of Entomology, Michigan State University, 202 CIPS, 578 Wilson Road, East Lansing, MI 48824
2. Department of Entomology, Cornell University, 4129 Comstock Hall, Ithaca, NY 14853
3. Current Affiliation: U.S. Department of Agriculture – Agricultural Research Service, Pollinating Insect – Biology, Management, Systematics Research Unit, 1410 N. 800 E., Logan, UT 84341

***Corresponding author:** Kelsey K. Graham, kelsey.katherine.graham@gmail.com, +1 435.797.3879

Supplemental figures and tables

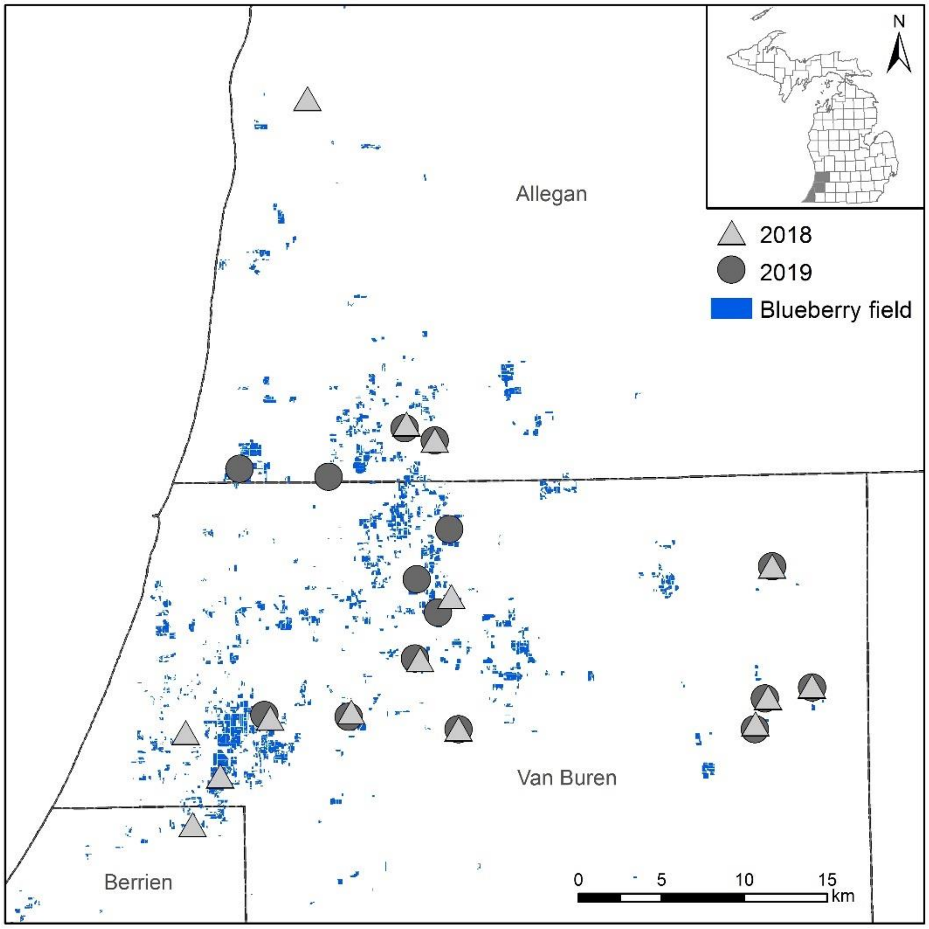

Figure S1. Locations of blueberry fields where pollen collected by honey bee and bumble bee colonies was sampled for pesticide residues in 2018 and 2019. Blue patches indicate the location of blueberry fields, determined by analysis of aerial imagery. Map created using ArcGIS® software by Esri. ArcGIS® and ArcMap™ are the intellectual property of Esri and are used under license. Copyright © Esri. All rights reserved. For more information about Esri® software, please visit www.esri.com. Basemap data sources: ArcUSA, U.S. Census, USDA, and Esri.

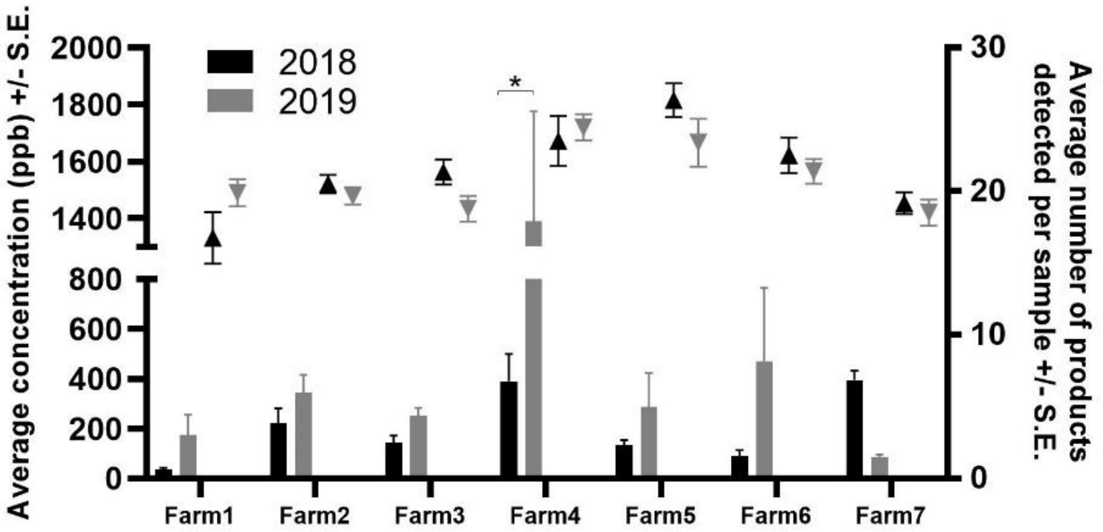

Figure S2. Comparison of average total pesticide concentrations in pollen samples (bars) and average number of active ingredients detected per sample (triangles) for pollen collected by honey bees at blueberry farms during the bloom period of two growing seasons. Graph created in GraphPad Prism 9[1].

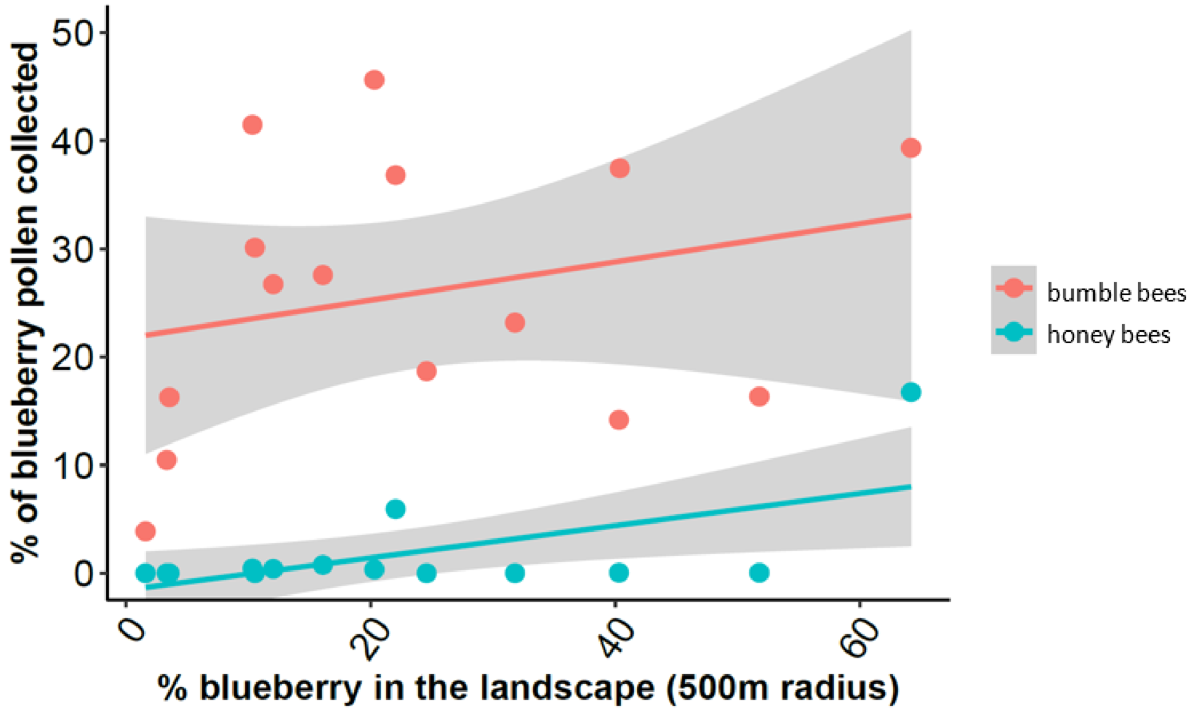

Figure S3. Correlation between percentage of land used for blueberry production within 500m of bee colonies, and the amount of blueberry pollen collected. Each dot represents the average blueberry pollen collected at a farm. Lines indicate smoothed linear regression lines (function: geom_smooth(method=lm), package: ggplot2[2]) and grey area indicates the 95% confidence interval. Graph created in R[3] v3.6.2 with the package ggplot2[2].

Table S1. Active ingredients detected in bee collected pollen samples in 2018 and 2019, ordered by percent of samples with detections. Shaded cells indicate active ingredients that were found in over 90% of samples.

						2018				2019							
						Honey bee collected pollen				Bumble bee collected pollen				Honey bee collected pollen			
**Active ingredient**	**Type**	**Registered on blueberries**	**Applied to sampled fields**	**Applied to blueberry during bloom**	**Applied to honey bees**	**Mean (ppb)**	**StDev**	**% of samp. with detects**	**Max (ppb)**	**Mean (ppb)**	**StDev**	**% of samp. with detects**	**Max (ppb)**	**Mean (ppb)**	**StDev**	**% of samp. with detects**	**Max (ppb)**
atrazine	H					10.95	5.79	100	23.69	7.63	13.65	100	55.68	9.40	35.55	100	320.48
metolachlor	H	x	x	x		7.88	8.98	100	54.34	7.85	8.53	100	30.33	8.05	27.30	100	246.71
azoxystrobin	F	x	x	x		32.93	83.28	99	585.79	44.39	99.81	100	390.54	15.99	53.54	100	385.15
fluopyram	F	x	x	x		1.73	2.98	99	15.96	69.43	107.13	100	322.16	43.69	171.52	100	1365.93
boscalid	F	x	x	x		38.50	79.17	96	375.29	351.63	569.58	100	1757.59	91.90	339.30	94	2479.06
imidacloprid	I	x	x			1.01	1.05	99	8.71	0.69	0.54	93	2.36	0.54	0.26	91	1.99
chlorpyrifos	I					3.04	4.34	76	27.10	22.22	69.60	100	272.69	14.60	40.43	97	213.47
pyraclostrobin	F	x	x	x		21.47	42.92	95	188.11	139.55	213.70	87	588.58	52.03	161.08	91	1204.12
carbendazim	F					19.86	20.14	88	89.15	6.87	1.97	80	13.11	216.32	713.00	95	5753.56
fenbuconazole	F	x	x	x		12.37	14.56	72	59.20	177.40	230.57	100	636.60	5.84	10.74	87	72.09
methoxyfenozide	I	x	x	x		26.14	65.85	89	285.96	285.93	488.13	80	1406.19	28.13	59.14	73	277.63
difenoconazole	F					1.81	4.86	86	34.91	2.72	3.94	87	15.22	2.32	7.90	70	58.09
propiconazole	F	x	x	x		15.27	20.54	74	83.49	83.27	89.33	87	280.93	15.62	41.44	71	279.36
thiophanate methyl	F					0.61	0.44	64	2.34	0.67	0.27	100	1.07	2.97	12.51	54	89.06
cyprodinil	F	x				3.48	7.10	74	48.36	5.10	7.74	80	28.31	5.02	11.41	60	58.74
clothianidin	I					2.25	3.99	82	27.29	1.98	0.84	60	3.18	2.23	5.52	68	35.72
metconazole	F	x	x	x		9.53	16.45	62	99.29	24.24	30.96	60	92.31	13.67	43.77	63	333.01
carbaryl	I	x				4.89	10.58	87	56.81	2.50	2.59	47	7.68	6.00	16.44	37	93.15
diuron	H	x	x	x		1.19	0.47	29	2.42	39.39	98.53	93	375.23	18.32	74.60	41	475.60
pyrimethanil	F	x	x	x		4.72	7.32	45	32.63	162.50	178.92	40	406.09	136.22	350.61	57	2071.38
myclobutanil	F					1.56	2.53	61	17.25	17.28	20.86	20	40.61	1.39	2.86	56	19.95
amitraz	M				x	3.51	5.02	13	14.09	1.09	0.51	53	2.24	21.07	48.45	60	267.01

propazine	H					0.31	0.07	67	0.51	0.48	0.39	20	0.92	0.59	1.15	25	5.69
eimethenamid	H					2.24	4.85	55	31.50	17.83	30.50	27	63.48	2.23	2.34	29	9.47
fluxapyroxad	F					0.45	0.09	41	0.83	0.68	0.26	27	0.94	1.23	1.73	29	8.93
trifloxystrobin	F					0.43	0.33	58	1.30	7.48		7	7.48	0.35	0.26	20	1.27
coumaphos	M				x	0.67	0.53	14	2.14					3.56	10.10	63	65.70
methoprotryne	H					0.31	0.24	18	1.02					0.57	0.69	56	3.90
piperonyl butoxide	S					0.29	0.06	12	0.44					4.31	14.42	61	85.20
methomyl	I	x	x	x		3.81	10.50	43	54.12	0.47	0.07	13	0.52	1.05	0.92	13	3.30
metalaxyl	F					3.12	7.06	24	24.65	1.54	1.82	13	2.83	5.32	10.65	23	33.07
thiamethoxam	I					1.07	0.64	46	4.24					2.17	3.80	13	14.74
acetamiprid	I	x	x			0.35	0.21	39	1.05					1.25	1.91	20	8.71
chlorantraniliprole	I					1.17	1.54	42	7.80					3.02	8.27	13	30.38
tebuconazole	F					1.83	0.44	12	3.00	3.61	2.72	13	5.53	2.47	1.91	29	10.48
azinphos methyl	I					7.59	5.91	17	23.20	22.87	26.41	13	41.55	10.98	9.23	5	22.66
clomazone	H					1.28	1.15	16	3.50	3.64		7	3.64	0.70	0.19	7	1.12
prometryn	H					0.22		1	0.22					0.23	0.03	16	0.33
thiobencarb	H													0.84	0.00	18	0.84
benoxacor	H					1.31	0.14	12	1.67					3.77	4.34	3	8.78
flutriafol	F					1.68	0.00	4	1.68	13.16		7	13.16			0	
spinosyn a	I					3.86	4.72	9	11.78								
isoxaben	H					0.48	0.57	8	1.64					0.25		1	0.25
spirotetramat	I					0.45	0.07	7	0.57					1.60	0.78	2	2.16
hexazinone	H	x	x											0.60	0.19	8	0.95
bromacil	H									1.26		7	1.26	1.26		1	1.26
indoxacarb	I					11.28	6.35	7	17.56					4.20		1	4.20
propamocarb	F													0.27	0.05	7	0.37
isoprothiolane	F					0.31		1	0.31					0.39	0.19	5	0.72
sethoxydim	H					0.86		1	0.86					0.82	0.35	5	1.26
fluoxastrobin	F					0.42	0.00	5	0.42					0.42		1	0.42
cyantraniliprole	I	x	x			1.26	0.00	4	1.26					1.60	0.48	2	1.94
isoxadifen ethyl	H					1.09	0.75	5	2.12								

isocarbophos	I													0.84	0.00	5	0.84
triadimenol	F													8.29	16.03	5	36.96
carbofuran	I					0.08		1	0.08					0.17	0.08	3	0.24
tebufenozide	I													9.01	15.98	4	32.99
avermectin b1a	I					4.20	0.00	3	4.20					7.18		1	7.18
buprofezin	I					0.25	0.00	3	0.25					0.74		1	0.74
diflubenzuron	I					2.30		1	2.30					1.26	0.00	2	1.26
ethoxyquin	ANT													5.32	1.94	3	7.56
difenacoum	R					0.42	0.00	3	0.42								
fludioxonil	F	x				4.64	0.63	3	5.09								
thiacloprid	I					0.63	0.08	3	0.69								
penthiopyrad	F					0.59		1	0.59					0.42		1	0.42
thiabendazole	F					0.42		1	0.42					0.42		1	0.42
dithiopyr	H													4.20	0.00	2	4.20
napropamide	H													1.17	0.35	2	1.41
azaconazole	F					0.25		1	0.25								
bromuconazole	F					0.84		1	0.84								
prometon	H					0.25		1	0.25								
spiromesifen	I					10.21		1	10.21								
tebuthiuron	H					0.21		1	0.21								
tetramethrin	I					0.84		1	0.84								
ancymidol	H													4.20		1	4.20
benzoximate	A													0.84		1	0.84
chlorfenvinphos	I													0.42		1	0.42
ethofumesate	H													10.68		1	10.68
fenpyroximate	A													0.25		1	0.25
malaoxon*	I	x	x											0.25		1	0.25
picoxystrobin	F													0.25		1	0.25
tebufenpyrad	I													0.42		1	0.42

* Breakdown product of malathion

Pesticide types: I=insecticide, F=fungicide, H=herbicide, M=miticide (in-hive treatment for Varroa), A=acaracide, S=synergist, Ant=antioxidant, R=rodenticide

Table S2. Retention times and optimized SRM acquisition parameters for pesticides and internal standards (RT: Retention time, CE: Collision Energy). Limit of Detection (LOD), Limit of Quantification (LOQ) and Upper Limit of Linearity (ULOL) are also included.

Compound	RT (min)	Polarity	Precursor (m/z)	RF Lens (V)	Quantifying ion (m/z)	CE 1 (V)	Confirmation ion (m/z)	CE 2 (V)	LOD (ppb)	LOQ (ppb)	ULOL (ppb)
d_7-propamocarb	3.25	Positive	196.2	97	103.1	18	151.2	14			
d_4-imidacloprid	5.05	Positive	260.1	114	213.1	16	179.1	19			
d_7-bentazone	6.42	Negative	246	134	132.1	26	182.2	20			
d_5-atrazine	7.42	Positive	221	113	179.1	18	101	25			
d_{10}-chlorpyrifos	9.66	Positive	359.9	123	199	199	98.9	31			
chlormequat chloride	0.73	Positive	122	113	58	27	63	21	0.21	0.63	350
mepiquat chloride	0.79	Positive	114.1	128	98	26	58	25	0.14	0.42	350
methamidophos	1.79	Positive	141.9	100	94	14	125	14	0.35	1.05	1400
cyromazine	2.4	Positive	167	133	85.1	19	125.1	18	1.4	4.2	1400
acephate	2.74	Positive	184	65	143	10	94.8	23	1.4	4.2	1400
omethoate	3.24	Positive	214	115	182.8	10	124.9	18	0.14	0.42	1400
propamocarb	3.28	Positive	189.1	98	102	17	74.1	25	0.084	0.252	1400
aminocarb	3.32	Positive	209.1	124	137.1	24	152.1	14	0.084	0.252	1400
formetanate hydrochloride	3.35	Positive	222.1	144	165.1	15	120	27	0.42	1.26	1400
butocarboxim sulfoxide	3.47	Positive	207	94	132	10	88	10	1.4	4.2	1400
pymetrozine	3.57	Positive	218	150	105	20	78.1	39	0.42	1.26	350
dinotefuran	3.59	Positive	203	98	113.1	10	129.1	12	0.42	1.26	1400
butoxycarboxim	3.65	Positive	223	124	166.1	15	46.1	26	1.4	4.2	1400
aldicarb sulfone	3.71	Positive	223.1	117	148	10	86.1	16	1.4	4.2	1400
oxamyl	3.9	Positive	237	73	72.1	10	90	10	0.14	0.42	1400
methomyl	4.13	Positive	163.1	71	87.9	10	106.1	10	0.14	0.42	1400
demeton-s-methylsulfone	4.25	Positive	263	150	169	16	108.9	28	0.14	0.42	1400
thiamethoxam	4.39	Positive	292	121	211.1	12	181	22	0.14	0.42	350
carbendazim	4.42	Positive	192	103	160.1	18	132	30	2.1	6.3	1400
mexacarbate	4.5	Positive	223.1	136	151.1	24	166.1	15	0.021	0.063	1400
monocrotophos	4.54	Positive	224	112	127	16	192.9	10	0.28	0.84	1400

ethiofencarb sulfone	4.73	Positive	258	119	107	16	201	10	0.7	2.1	1400
dicrotophos	4.81	Positive	238	127	127	18	192.5	10	0.14	0.42	1400
pirimicarb-desmethyl	4.84	Positive	225.2	135	168	15	72	21	0.084	0.252	1400
ethiofencarb sulfoxide	4.91	Positive	242	106	107	18	185	10	0.21	0.63	1400
trichlorfon	4.94	Positive	256.9	131	108.9	18	79	30	0.42	1.26	1400
clothianidin	5.03	Positive	250	104	169	13	131.9	17	0.28	0.84	350
imidacloprid	5.06	Positive	256	131	209	16	175.1	19	0.14	0.42	350
fenuron	5.13	Positive	164.8	116	72.1	15	46.1	15	0.42	1.26	350
thiabendazole	5.13	Positive	202	208	175	26	131	33	0.28	0.84	350
flumetsulam	5.17	Positive	326	192	129	26	262.1	19	1.4	4.2	1400
dimethoate	5.19	Positive	229.8	106	198.8	10	124.9	22	0.28	0.84	350
3-hydroxy-carbofuran	5.21	Positive	238.1	119	181	10	163.1	16	0.28	0.84	350
vamidothion	5.25	Positive	288	120	146	14	118.1	23	0.14	0.42	350
fuberidazole	5.26	Positive	185.1	152	157.1	22	129	35	0.28	0.84	1400
mevinphos	5.27/5.83	Positive	225	97	127	17	192.9	10	0.42	1.26	350
metamitron	5.29	Positive	203	170	174.8	17	104	23	1.4	4.2	350
methiocarb sulfoxide	5.29	Positive	242	134	185	14	122.1	29	0.14	0.42	350
chloridazon	5.41	Positive	222	152	104	23	92	26	0.14	0.42	350
acetamiprid	5.55	Positive	223	118	126	21	90	34	0.084	0.252	350
methiocarb sulfone	5.62	Positive	258	119	122	19	201	10	0.28	0.84	1400
schradan	5.69	Positive	287.1	144	242.1	14	135.1	26	0.07	0.21	1400
ethirimol	5.89	Positive	209.8	192	140.1	22	98	27	0.14	0.42	350
florasulam	5.96	Positive	360	179	129	25	108.9	53	1.4	4.2	1400
pirimicarb	5.97	Positive	239.1	147	182.1	16	72	21	0.14	0.42	350
thiacloprid	5.98	Positive	253	162	126	21	90	36	0.14	0.42	1400
metoxuron	6.21	Positive	229	145	72.1	18	156	26	0.28	0.84	1400
formothion	6.23	Positive	258	80	199	10	124.9	22	1.4	4.2	350
imazethapyr	6.29	Positive	290.1	189	177	27	248.1	19	0.14	0.42	350
carbetamide	6.33	Positive	237.1	102	192	10	120	16	0.14	0.42	350
metolcarb	6.34	Positive	166	83	108.9	10	94	31	0.28	0.84	350
oxadixyl	6.35	Positive	279.1	120	219.1	10	132.1	31	0.14	0.42	350

tricyclazole	6.45	Positive	190	178	163	23	136	29	0.028	0.084	350
bentazone	6.46	Negative	238.9	169	132	26	197	21	0.7	2.1	350
cyanazine	6.46	Positive	241.1	164	214.1	18	103.9	29	0.42	1.26	1400
azamethiphos	6.56	Positive	324.9	166	182.9	16	111.9	34	0.084	0.252	350
bromacil	6.6	Positive	261	112	204.9	14	187.8	28	0.42	1.26	350
propoxur	6.6	Positive	210	87	111	14	168.1	10	0.28	0.84	350
thiophanate-methyl	6.62	Positive	343	161	151	20	93	46	0.28	0.84	350
bendiocarb	6.67	Positive	224	104	167.1	10	108.9	18	0.42	1.26	350
carbofuran	6.68	Positive	222	111	165.1	12	123	22	0.028	0.084	350
ofurace	6.76	Positive	282.1	145	254.1	12	160.1	24	0.14	0.42	350
malaoxon	6.79	Positive	315	133	98.9	23	269	10	0.084	0.252	350
imazaquin	6.81	Positive	312.1	195	267.1	21	199	28	0.28	0.84	350
thidiazuron	6.83	Positive	220.6	119	101.9	16	127.9	17	0.28	0.84	350
pyroxsulam	6.83	Positive	435	262	195.1	26	258	22	0.42	1.26	350
simetryn	6.83	Positive	214.1	161	124.1	20	96	25	0.14	0.42	350
desmetryn	6.85	Positive	214.1	161	172	18	82	30	0.14	0.42	350
ancymidol	6.86	Positive	257.1	157	135	25	81.1	25	1.4	4.2	350
hexazinone	6.89	Positive	253.1	141	171.1	16	71.1	31	0.14	0.42	350
tebuthiuron	6.9	Positive	229	145	172.1	18	116	27	0.07	0.21	350
metosulam	6.99	Positive	418	233	174.9	27	140	50	5.6	16.8	350
prometon	7.08	Positive	226.1	156	184.1	19	142.1	23	0.084	0.252	350
carbaryl	7.08	Positive	202	95	145.1	10	127	29	0.28	0.84	1400
fenthion sulfoxide	7.09	Positive	295	187	280	19	108.9	32	0.14	0.42	1400
ethiofencarb	7.09	Positive	226.1	105	107	17	164.1	10	4.2	12.6	350
cyantraniliprole	7.1	Positive	475	158	285.9	11	444	18	0.42	1.26	350
terbumeton	7.2	Positive	226.2	153	170	17	142.1	23	2.8	8.4	1400
monolinuron	7.2	Positive	215	131	126	18	148	15	0.14	0.42	1400
fosthiazate	7.21	Positive	284	118	103.9	21	228	10	0.14	0.42	1400
fluometuron	7.24	Positive	233	145	72	19	46	18	0.28	0.84	1400
2,4-d	7.27	Negative	218.9	101	160.9	13	125	26	140	420	1400
bromoxynil	7.29	Negative	275.8	194	80.9	31	78.9	30	4.2	12.6	350

dnoc	7.3	Negative	197	147	180	19	137	18	2.8	8.4	1400
ethoxyquin	7.36	Positive	218.1	183	160.1	33	148.1	22	1.4	4.2	350
benodanil	7.36	Positive	323.8	180	231	23	202.9	35	0.084	0.252	1400
imazalil	7.37	Positive	297	170	156	23	200.9	18	0.14	0.42	350
isoprocarb	7.38	Positive	194.1	105	95	15	137.1	10	0.28	0.84	1400
flutriafol	7.42	Positive	302	144	70	19	123	28	0.56	1.68	1400
chlorotoluron	7.43	Positive	213	141	72.1	18	46	16	0.28	0.84	1400
atrazine	7.44	Positive	216.1	167	174	18	103.9	28	0.21	0.63	1400
metobromuron	7.47	Positive	258.9	114	148	15	169.9	19	0.14	0.42	1400
metazachlor	7.48	Positive	278	111	210.1	10	134.1	22	0.084	0.252	350
lenacil	7.5	Positive	235.1	109	153.1	16	136	32	0.28	0.84	1400
isocarbophos	7.53	Positive	307	73	231	16	273	10	0.28	0.84	1400
metalxyl	7.54	Positive	280.1	98	220	14	192.2	18	0.084	0.252	1400
griseofulvin	7.54	Positive	353.2	188	285	18	165.1	20	0.14	0.42	1400
methoprotryne	7.59	Positive	272.1	175	240.2	19	198	23	0.07	0.21	350
isoproturon	7.59	Positive	207.1	143	72.1	19	165.1	14	0.28	0.84	1400
fensulfothion	7.64	Positive	309	180	280.9	15	253	18	0.084	0.252	1400
heptenophos	7.69	Positive	251	123	127	17	124.9	13	0.42	1.26	350
desmedipham	7.71	Positive	301.3	133	182	10	136	20	0.42	1.26	1400
forchlorfenuron	7.71	Positive	248	134	129	18	93	33	0.14	0.42	1400
dodemorph	7.73	Positive	282.2	188	116.1	21	98	27	0.14	0.42	350
cycluron	7.73	Positive	199.1	137	72.1	22	69.1	21	0.14	0.42	1400
chlorantraniliprole	7.75	Positive	481.9	182	283.9	12	450.8	18	0.14	0.42	1400
methabenzthiazuron	7.75	Positive	222	118	165.1	17	150	33	0.056	0.168	1400
diuron	7.77	Positive	233	145	72.1	19	46	18	0.28	0.84	1400
ioxynil	7.79	Negative	369.7	204	126.8	35	214.9	32	1.4	4.2	350
azaconazole	7.82	Positive	299.9	162	159	28	231	17	0.084	0.252	350
phenmedipham	7.82	Positive	301.1	145	168	10	136	20	4.2	12.6	1400
dimefuron	7.83	Positive	339	220	167	22	72.1	26	0.28	0.84	350
benoxacor	7.84	Positive	260	173	149.1	18	134	29	0.42	1.26	1400
clomazone	7.91	Positive	240	134	125	21	89	47	0.21	0.63	1400

diethofencarb	7.92	Positive	268	114	226.1	10	124	32	0.14	0.42	350
azinphos-methyl	7.93	Positive	317.9	103	132	15	125	17	1.4	4.2	1400
fenobucarb	7.93	Positive	208.1	110	95	15	152	10	0.14	0.42	350
ethofumesate	7.98	Positive	287.1	159	121	16	259.1	10	2.8	8.4	350
fluazifop	8.01	Positive	328	174	282	19	254	26	0.42	1.26	350
azoxystrobin	8.03	Positive	404.1	175	372	14	344.1	25	0.084	0.252	350
propazine	8.03	Positive	230.1	177	146.1	23	188.1	18	0.084	0.252	1400
pyrimethanil	8.03	Positive	200.1	184	107	25	168.1	30	0.28	0.84	350
nuarimol	8.04	Positive	315.1	177	252.1	22	243	25	1.4	4.2	350
ethiprole	8.04	Positive	396.9	189	350.9	21	255	36	0.28	0.84	350
fenamidone	8.05	Positive	312.1	151	236.1	15	92	25	0.14	0.42	350
halofenozide	8.07	Positive	331	99	275.1	10	105	18	0.28	0.84	350
dimethenamid	8.09	Positive	276.1	135	244.1	14	168.1	24	0.28	0.84	1400
prometryn	8.11	Positive	242.2	149	158	24	200	19	0.07	0.21	350
methiocarb	8.13	Positive	226.1	105	169.1	10	121	19	0.42	1.26	1400
spiroxamine	8.18	Positive	298.2	167	144.2	20	100	30	0.14	0.42	350
crotoxyphos	8.18	Positive	332	100	210.9	10	127	25	0.14	0.42	350
mandipropamid	8.18	Positive	412.1	186	328.1	15	356.1	10	0.28	0.84	1400
terbuthylazine	8.19	Positive	230.1	140	174	17	132	25	0.28	0.84	350
boscalid	8.2	Positive	343	174	307	21	272	30	0.14	0.42	350
isoxaben	8.2	Positive	333.2	167	164.9	19	150	39	0.084	0.252	350
promecarb	8.21	Positive	208.1	107	109	16	151.1	10	0.14	0.42	350
paclobutrazol	8.22	Positive	294	151	70.1	21	125	38	0.28	0.84	1400
terbutryn	8.23	Positive	242.1	158	186	19	91	28	0.42	1.26	350
fluopicolide	8.23	Positive	382.9	193	172.9	23	144.9	48	0.14	0.42	1400
propyzamide	8.26	Positive	256	110	190	14	173	29	0.14	0.42	350
mepronil	8.27	Positive	270.1	159	118.9	24	228.1	15	0.084	0.252	350
fluxapyroxad	8.28	Positive	382	140	362.1	13	342.1	20	0.14	0.42	350
fludioxonil	8.28	Negative	247	149	180	28	126.1	31	1.4	4.2	350
isoprothiolane	8.32	Positive	291.1	116	231	10	188.8	22	0.084	0.252	1400
methoxyfenozide	8.32	Positive	369.2	113	149.1	17	313.1	10	0.14	0.42	1400

dimethomorph	8.33	Positive	388.1	225	301	21	165.1	32	0.28	0.84	350
triadimefon	8.34	Positive	294	138	197	16	141	22	0.084	0.252	1400
propetamphos	8.34	Positive	282	106	138	17	156	10	1.4	4.2	1400
myclobutanil	8.39	Positive	289	121	70.1	18	124.9	33	0.14	0.42	350
fluorochloridone	8.4	Positive	312	132	292	21	145	48	1.4	4.2	350
butafenacil	8.42	Positive	492.1	170	331	24	349	15	0.084	0.252	1400
cumyluron	8.43	Positive	303.1	137	184.9	13	125	33	0.084	0.252	1400
fluopyram	8.43	Positive	397	202	208	22	173	29	0.084	0.252	1400
iprovalicarb	8.44	Positive	321.2	129	119.1	19	116.1	20	1.4	4.2	1400
fenhexamid	8.45	Positive	302	166	97	23	55	35	1.4	4.2	350
bifenazate	8.46	Positive	301.1	119	198	10	170.1	19	0.14	0.42	350
fluoxastrobin	8.49	Positive	459.1	219	427	17	188	35	0.14	0.42	350
triazophos	8.49	Positive	314	164	162.1	19	119	34	0.084	0.252	350
bromuconazole	8.49/8.90	Positive	377.8	185	159	30	161	31	0.28	0.84	350
mefenacet	8.49	Positive	299	132	148.1	14	120	25	0.07	0.21	1400
spirotetramat	8.5	Positive	374.1	185	302.1	17	330.2	15	0.14	0.42	1400
bupirimate	8.5	Positive	317.1	204	166.1	21	272.1	20	0.14	0.42	350
fluquinoconazole	8.5	Positive	375.9	130	349	19	307.1	26	0.28	0.84	1400
flufenacet	8.51	Positive	364	126	194	10	152.1	19	0.07	0.21	350
tepraloxydim	8.52	Positive	342.1	153	250.2	13	166.1	21	2.8	8.4	1400
simeconazole	8.53	Positive	294.1	150	70.1	20	135.1	21	0.56	1.68	1400
chloroxuron	8.54	Positive	291	178	72.1	21	46	19	0.7	2.1	1400
tetraconazole	8.56	Positive	372	187	158.9	30	123	55	0.28	0.84	350
dimethametryn	8.58	Positive	256.1	180	185.9	21	96	30	0.28	0.84	350
trietazine	8.58	Positive	230.1	178	132	22	104	29	0.28	0.84	1400
cyazofamid	8.63	Positive	325	122	107.9	14	217	18	0.28	0.84	350
napropamide	8.65	Positive	272	149	171.1	19	199	13	0.084	0.252	350
alachlor	8.64	Positive	270.1	112	238.1	10	162.2	20	0.7	2.1	1400
metolachlor	8.64	Positive	284.1	143	252.1	15	176.1	26	0.084	0.252	1400
fipronil	8.65	Negative	434.9	138	330	15	250	26	0.84	2.52	87.5
epoxiconazole	8.68	Positive	330	149	121	21	100.9	44	0.28	0.84	1400

fenbuconazole	8.7	Positive	337.1	188	125	31	70.1	21	0.28	0.84	1400
fenamiphos	8.72	Positive	304.1	180	217	18	201.9	35	0.14	0.42	1400
haloxyfop	8.72	Positive	361.8	166	316	18	91	30	1.4	4.2	350
picoxystrobin	8.72	Positive	368.1	111	145.1	21	205	10	0.084	0.252	350
tebufenozide	8.73	Positive	353.2	110	297.1	10	133.1	19	0.28	0.84	350
triadimenol	8.73	Positive	297	101	133	14	105	39	0.35	1.05	350
flubendiamide	8.74	Positive	683	195	407.9	10	274	30	1.4	4.2	1400
rotenone	8.74	Positive	395.1	231	213.1	23	192.1	24	0.28	0.84	1400
fenoxycarb	8.75	Positive	302	152	88	19	116	10	0.14	0.42	350
flusilazole	8.76	Positive	316.1	192	247.1	18	165.1	27	0.14	0.42	1400
carfentrazone-ethyl	8.76	Positive	412	217	346	23	365.9	18	0.7	2.1	1400
diflubenzuron	8.76	Positive	311	131	158	13	141.1	32	0.42	1.26	350
dimoxystrobin	8.77	Positive	327.2	108	204.9	10	115.9	22	0.084	0.252	1400
phenthoate	8.78	Positive	320.8	119	247.1	12	135	20	0.28	0.84	350
isoxadifen-ethyl	8.79	Positive	296.1	146	232.1	17	263.2	10	0.14	0.42	350
kresoxim-methyl	8.79	Positive	314.1	116	267.1	10	222.1	13	0.28	0.84	1400
neburon	8.8	Positive	275	171	88.1	16	57	21	0.28	0.84	1400
sulfotep	8.81	Positive	323	145	171	14	114.9	29	0.14	0.42	1400
penthiopyrad	8.84	Positive	360.1	149	276	14	256.1	20	0.14	0.42	1400
fipronil sulfone	8.86	Negative	450.8	152	415	15	282	26	0.28	0.84	350
tebuconazole	8.87	Positive	308	162	70.1	23	125	38	0.56	1.68	1400
cyprodynil	8.88	Positive	226.1	153	93	34	108.1	26	0.28	0.84	350
anilofos	8.89	Positive	368	173	198.9	14	124.9	31	0.28	0.84	1400
carpropamid	8.9	Positive	334	126	139	20	195.9	13	0.28	0.84	350
etrimfos	8.9	Positive	293	178	265.1	17	124.9	26	0.28	0.84	350
chlorfenvinphos	8.93	Positive	359	159	155.1	13	169.9	39	0.14	0.42	350
penconazole	8.93	Positive	284.1	134	159	30	70.1	18	0.28	0.84	1400
zoxamide	8.95	Positive	336	167	186.9	22	159	39	0.28	0.84	1400
benzoylprop-ethyl	8.95	Positive	366	134	105	16	77.1	48	0.14	0.42	1400
fenthion	8.96	Positive	279	133	169	16	247	13	0.84	2.52	1400
cyflufenamid	8.99	Positive	413	161	295	15	241.1	23	0.084	0.252	1400

propiconazole	8.99	Positive	342.1	69	159	30	122.9	55	0.7	2.1	350
pirimiphos-methyl	9	Positive	306.1	199	164.1	22	108	31	0.14	0.42	1400
coumaphos	9.01	Positive	362.9	169	227	26	306.8	18	0.14	0.42	350
hexaconazole	9.01	Positive	314	151	70	21	159	32	1.4	4.2	1400
metconazole	9.03	Positive	320.1	170	70.1	24	125	38	0.84	1.68	350
phoxim	9.04	Positive	299	95	129	11	77	30	0.14	0.42	350
pyraclostrobin	9.04	Positive	387.8	159	194	12	163.1	24	0.14	0.42	350
benzoximate	9.06	Positive	364.1	105	199	10	105	24	0.28	0.84	350
prochloraz	9.07	Positive	376	125	307.9	10	266	17	0.35	1.05	350
spinosad (spinosyn a)	9.09	Positive	732.4	299	142.1	29	98.1	45	0.35	1.05	1400
metrafenone	9.1	Positive	409	169	209.1	14	227	21	0.28	0.84	350
pencycuron	9.12	Positive	329.1	190	125	40	218.1	16	0.14	0.42	350
haloxyfop-methyl	9.15	Positive	376	180	315.8	17	91	31	0.112	0.336	350
thiobencarb	9.15	Positive	258	118	125	20	89	49	1.4	4.2	350
indoxacarb	9.16	Positive	528	233	203	38	150	24	1.4	4.2	1400
diniconazole	9.16	Positive	326	178	70	26	159.1	31	1.4	4.2	1400
trifloxystrobin	9.18	Positive	409	179	186	18	145	44	0.084	0.252	350
piperophos	9.19	Positive	354.1	175	171	22	255	14	0.084	0.252	1400
difenoconazole	9.24	Positive	406	214	251	26	337	18	0.14	0.42	1400
dithiopyr	9.24	Positive	402	167	354	18	272	29	1.4	4.2	350
cycloate	9.25	Positive	216	126	154.1	12	83.1	16	0.7	2.1	1400
hexaflumuron	9.28	Negative	458.8	142	438.9	10	175	36	2.8	8.4	1400
clethodim	9.27	Positive	360.1	140	164.1	18	268.1	12	0.14	0.42	1400
prosulfocarb	9.34	Positive	252.1	139	91	22	128.1	13	0.084	0.252	350
triflumizole	9.34	Positive	346	113	278.1	10	73.1	17	0.084	0.252	350
furathiocarb	9.38	Positive	383.1	176	194.9	18	252.1	13	0.28	0.84	1400
quizalofop-ethyl	9.39	Positive	373	221	299	19	271.1	26	0.28	0.84	350
buprofezin	9.4	Positive	306.1	129	201.1	12	116	16	0.084	0.252	350
profenophos	9.41	Positive	374.9	171	304.8	19	346.9	13	0.14	0.42	350
tetramethrin	9.41	Positive	332.2	139	164.1	24	135.1	18	0.28	0.84	1400
sethoxydim	9.43	Positive	328.2	153	178	19	282.1	12	0.14	0.42	350

fluazinam	9.44	Negative	462.8	194	415.9	19	398	16	0.42	1.26	1400
tebufenpyrad	9.44	Positive	334.2	206	117	36	145	27	0.14	0.42	350
esprocarb	9.47	Positive	266.2	139	91	24	71.1	15	0.14	0.42	350
piperonyl butoxide	9.51	Positive	356.3	118	177.1	10	119	33	0.084	0.252	1400
tolfenpyrad	9.58	Positive	384.1	188	197	25	196	19	0.14	0.42	350
imibenconazole	9.54	Positive	411	209	125	31	171	10	0.56	1.68	1400
hexythiazox	9.63	Positive	353.1	123	228	15	168	25	0.28	0.84	1400
tralkoxydim	9.64	Positive	330.2	159	284.2	13	138.1	20	5.6	16.8	350
chlorpyrifos	9.66	Positive	349.9	126	197.9	19	321.7	11	0.28	0.84	350
spiromesifen	9.68	Positive	371.1	132	273.2	10	255.2	23	2.8	8.4	1400
flufenoxuron	9.69	Positive	489.2	142	158.1	18	140.9	41	0.14	0.42	1400
sulprofos	9.69	Positive	323	118	218.9	16	247	12	0.28	0.84	1400
etoxazole	9.74	Positive	360.1	201	141	31	304	18	0.084	0.252	1400
quinoxyfen	9.81	Positive	308	234	196.9	32	162	45	2.8	8.4	1400
chlorfluazuron	9.82	Positive	541.8	223	384.9	21	158	20	1.4	4.2	1400
difenacoum	9.84	Positive	445.1	236	179	31	257.2	20	0.14	0.42	1400
amitraz	9.87	Positive	294.1	103	163.1	14	122.1	29	0.28	0.84	1400
fenpyroximate	9.88	Positive	422.2	197	366.2	16	214.1	30	0.084	0.252	1400
avermectin b1a	9.95	Positive	890.5	225	305.1	25	567.2	14	1.4	4.2	1400
resmethrin	10.03	Positive	339.1	158	171.1	15	128	41	0.35	1.05	1400
brodifacoum	10.12	Positive	523.1	289	335	22	178	34	0.42	1.26	1400
fenazaquin	10.24	Positive	307.1	159	161.2	17	57.1	23	0.14	0.42	1400
etofenprox	10.25	Positive	394	133	177.1	15	359.2	10	0.084	0.252	350

Table S3. Average and S.E. values for the (A) number of pesticide active ingredients (AIs) in samples and (B) average and S.E. concentrations (in ppb) of pesticides in pollen samples collected from returning honey bee and bumble bee foragers in blueberry farms in 2018 and 2019. Data are broken down by AIs sprayed in the focal fields, registered for use on blueberries during bloom but not sprayed in the focal fields, and not registered for use on blueberry during bloom (could be AIs that persisted from sprays on the focal farm outside bloom, or from other crops). Maximum number of AIs and maximum concentrations are maximums from individual samples. Percentages are the average contribution of each likely pesticide source category to the overall sample AI count or concentration.

A. Active ingredients detected in pollen samples

Species	Year	Field mgmt.	AIs sprayed in the focal field during bloom			Sprayed on blueberry during bloom but not from focal field			Not registered for use on blueberry during bloom		
			Avg. AIs	Max. No. AIs	Avg. % of AIs	Avg. No. AIs	Max. No. AIs	Avg. % of AIs	Avg. No. AIs	Max. No. AIs	Avg. % of AIs
Honey bee	2018	Unsprayed	0		0	7.4 ± 0.5	10	34.6 ± 1.5	13.8 ± 0.7	21	65.4 ± 1.5
		Organic	0		0	9.8 ± 0.2	11	37.7 ± 0.7	16.2 ± 0.4	20	62.3 ± 0.7
		Conventional	2.3 ± 0.3	7	10.9 ± 1.3	6.5 ± 0.3	9	30.4 ± 1.1	12.8 ± 0.5	19	58.9 ± 0.9
Honey bee	2019	Unsprayed	0		0	8.3 ± 0.2	10	41.3 ± 1.2	12.2 ± 0.6	20	58.7 ± 1.2
		Conventional	2.8 ± 0.1	5	14.4 ± 0.9	6.2 ± 0.2	10	30.0 ± 1.0	11.5 ± 0.4	19	55.6 ± 0.7
Bumble bee	2019	Unsprayed	0		0	8.8 ± 0.6	10	51.0 ± 5.4	9.0 ± 1.8	16	48.9 ± 5.4
		Conventional	3.2 ± 0.3	5	16.7 ± 1.7	6.6 ± 0.5	9	34.8 ± 2.5	9.3 ± 0.4	11	48.5 ± 1.5

B. Concentration of pesticides in pollen samples

Species	Year	Field management	AIs sprayed in the focal field during bloom			Sprayed on blueberry during bloom but not from focal field			Not registered for use on blueberry during bloom		
			Avg. conc.	Max. conc.	Avg. % of total	Avg. conc	Max. conc.	Avg. % of total	Avg. conc.	Max. conc.	Avg. % of total
Honey bee	2018	Unsprayed	0		0	30.4 ± 7.6	87.1	36.3 ± 5.4	49.8 ± 9.0	89.2	63.7 ± 5.4
		Organic	0		0	128.2 ± 28.5	285.4	64.9 ± 3.8	47.5 ± 6.6	52.1	35.1 ± 3.8
		Conventional	130.9 ± 34.3	375.3	34.4 ± 6.2	105.2 ± 25.8	585.8	44.0 ± 5.9	48.8 ± 6.3	85.4	22.6 ± 2.7
Honey bee	2019	Unsprayed	0		0	69.9 ± 26.7	569.6	33.0 ± 3.9	203.4 ± 74.2	2333.2	67.0 ± 3.9
		Conventional	362.3 ± 96.8	2479.1	43.7 ± 4.1	115.0 ± 20.6	475.6	24.3 ± 3.0	272.3 ± 105.2	5753.6	32.0 ± 3.5
Bumble bee	2019	Unsprayed	0		0	233.7 ± 117.0	554.5	73.8 ± 16.4	90.3 ± 69.1	272.7	26.2 ± 16.4
		Conventional	1093.6 ± 204.4	1757.6	64.0 ± 7.4	572.2 ± 143.8	1406.2	33.6 ± 7.4	37.5 ± 13.0	63.5	2.4 ± 0.7

Supplemental methods:

Modified EN 15662 QuEChERS procedure for quantifying pesticide residues

Five grams of sample (or lower when insufficient volumes were obtained) were mixed with 7 mL of acetonitrile and 5 mL of water and then homogenized for 1 min using ceramic beads (2.8 mm diameter) and a Bead Ruptor 24 (OMNI International, USA). After complete homogenization, 6.5 g of EN 15662 salts were added (4 g MgSO4; 1 g NaCl; 1 g sodium citrate tribasic dihydrate; 0.5 g sodium citrate dibasic sesquihydrate). Samples were then thoroughly vortexed and centrifuged at 7300 × g for 5 minutes. One milliliter of supernatant was collected and transferred into a d-SPE (dispersive solid phase extraction) tube containing 150 mg $MgSO_4$ and 25 mg PSA. After the d-SPE step, 496 µL of supernatant were collected and 4 µL of internal standard solution (d_4-imidacloprid 0.07 ng/µL; d_{10}-chlorpyrifos 0.2 ng/µL: d_7-bentazon 0.1 ng/µL; d_5-atrazine 0.02 ng/µL; d_7-propamocarb 0.1 ng/µL) was added. The samples were then filtered (0.22 µm, PTFE) and stored at −20°C before analysis.

Sample analysis was performed with a Vanquish Flex UHPLC system (Dionex Softron GmbH, Germering, Germany) coupled with a TSQ Quantis mass spectrometer (Thermo Scientific, San Jose, CA). The UHPLC was equipped with an Accurcore aQ column (100 mm × 2.1 mm, 2.6 µm particle size). The mobile phase consisted of (A) Methanol/Water (2:98, v/v) with 5 mM ammonium formate and 0.1% formic acid and (B) Methanol/Water (98:2, v/v) with 5 mM ammonium formate and 0.1% formic acid. The column was maintained at a temperature of at 25°C throughout the run and the flow rate was set at 300 µL/min. The solvent gradient went as follows: 1.5 min equilibration (0% B) prior to injection, 0-0.5 min (0% B, isocratic), 0.5-7 min (0%-70% B, linear gradient), 7-9 min (70%-100% B, linear gradient), 9-12 min (100% B,

column wash), 12-12.1 min (100%-0% B, linear gradient), 12.1-14.5 min (0% B, re-equilibration). The flow from the LC was directed to the mass spectrometer through a Heated Electrospray probe (H-ESI). The settings of the H-ESI were: spray voltage 3700 V for positive mode and 2500 V for negative mode, sheath gas 40 (arbitrary unit), auxiliary gas 10 (arbitrary unit), sweep gas 1.5 (arbitrary unit), ion transfer tube temperature 325°C, and vaporizer temperature 350°C.

The MS/MS detection was carried out using the Selected Reaction Monitoring (SRM) mode. For each compound two transitions were monitored: one for quantification and the other for confirmation. The SRM parameters for each individual pesticide are summarized in Table S2. The resolution of both Q1 and Q3 was set at 0.7 FWHM, the cycle time was 0.5 s and the pressure of argon in the collision cell was set at 2 mTorr.

Works cited

1. GraphPad Software. GraphPad Prism. (2017).
2. Wickham, H. ggplot2: Elegant Graphics for Data Analysis. (2009).
3. R Core Team. R: A language and environment for statistical computing. (2019).
